# Myelinating satellite oligodendrocytes are integrated in a glial syncytium constraining neuronal high-frequency activity

**DOI:** 10.1038/ncomms11298

**Published:** 2016-05-10

**Authors:** Arne Battefeld, Jan Klooster, Maarten H. P. Kole

**Affiliations:** 1 Axonal Signalling Group, Netherlands Institute for Neuroscience, Royal Academy of Arts and Sciences, Meibergdreef 47, 1105 BA Amsterdam, The Netherlands; 2 Cell Biology, Department of Biology, Faculty of Science, University of Utrecht, 3584 CH Utrecht, The Netherlands

## Abstract

Satellite oligodendrocytes (s-OLs) are closely apposed to the soma of neocortical layer 5 pyramidal neurons but their properties and functional roles remain unresolved. Here we show that s-OLs form compact myelin and action potentials of the host neuron evoke precisely timed Ba^2+^-sensitive K^+^ inward rectifying (Kir) currents in the s-OL. Unexpectedly, the glial K^+^ inward current does not require oligodendrocytic Kir4.1. Action potential-evoked Kir currents are in part mediated by gap–junction coupling with neighbouring OLs and astrocytes that form a syncytium around the pyramidal cell body. Computational modelling predicts that glial Kir constrains the perisomatic [K^+^]_o_ increase most importantly during high-frequency action potentials. Consistent with these predictions neurons with s-OLs showed a reduced probability for action potential burst firing during [K^+^]_o_ elevations. These data suggest that s-OLs are integrated into a glial syncytium for the millisecond rapid K^+^ uptake limiting activity-dependent [K^+^]_o_ increase in the perisomatic neuron domain.

## Abstract (web summary)

Satellite oligodendrocytes (s-OLs) are characterised by their close proximity to neocortical pyramidal cells. Here, the authors find that s-OLs myelinate axons and activity of host neurons evokes inward K^+^ currents in s-OLs which may work to modulate action potential burst firing by buffering extracellular K^+^ levels.

## Graphical abstract

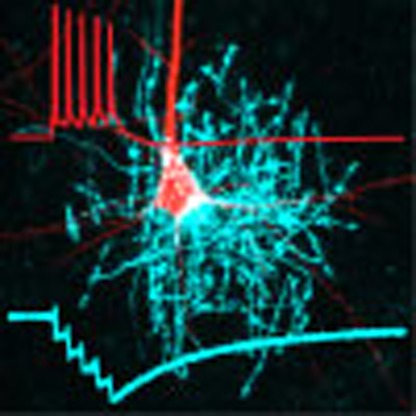

Neuron–glia interactions are critical for diverse functions of the central nervous system. Recent work shows that activity of glia modulates long-term synaptic potentiation or depression^1^,^2^,^3^, controls rhythmic network activity^4^ and contributes to neuronal dysfunction^5^. A tight organization of the glial syncytium around neurons has previously been observed to contribute to regulation of neuronal activity^6^,^7^, suggesting essential functional roles of local glia arrangements. In the grey matter, all glia cell types, including astrocytes and microglia, can be found in a satellite position around neurons but oligodendrocytes (OLs) are most frequently observed^8^,^9^,^10^. These OLs are referred to as perineuronal or satellite OLs (s-OLs)^9^,^11^,^12^,^13^. The present view of s-OLs is that they are primarily non-myelinating^10^,^12^,^14^,^15^ in contrast to the main function of OLs as myelin-producing cells^16^. However, s-OLs may provide metabolic support for neurons^10^, protect neurons against apoptosis^17^ or remyelinate axons following demyelinating injuries^12^. Since s-OLs are often located at the base of the soma^13^, close to the action potential (AP) initiation site at the axon initial segment (AIS)^18^, we here hypothesized that their privileged position enables s-OLs to influence AP firing.

To investigate the functional and anatomical properties of s-OLs, we combine simultaneous whole-cell patch-clamp recordings and live-confocal imaging with *post hoc* immunofluorescence and electron microscopy of neuron–s-OL pairs in acute neocortical slices from adult mice. We find that s-OLs myelinate surrounding axons and exhibit time-locked Ba^2+^- and carbenoxolone-sensitive inward currents in response to APs generated in the host neurons. Unexpectedly, the AP-evoked inwardly rectifying K^+^ (Kir) currents are not mediated by somatically expressed OL-specific Kir4.1 channels. Instead, the AP-evoked currents reflect junctional coupling to nearby astrocytes forming together with s-OLs a perisomatic glial syncytium. Moreover, during repetitive high-frequency AP firing of pyramidal neurons, s-OL coupled syncytia constrain the neuronal AP generation during periods of fast accumulation of [K^+^]_o_. These results suggest that s-OLs within a glial syncytium perform multiple functional and anatomical roles, spatially buffering K^+^ and myelinating axons within the perisomatic domain.

## Results

### Distribution of s-OLs around neocortical layer 5 neurons

**Figure 1:**
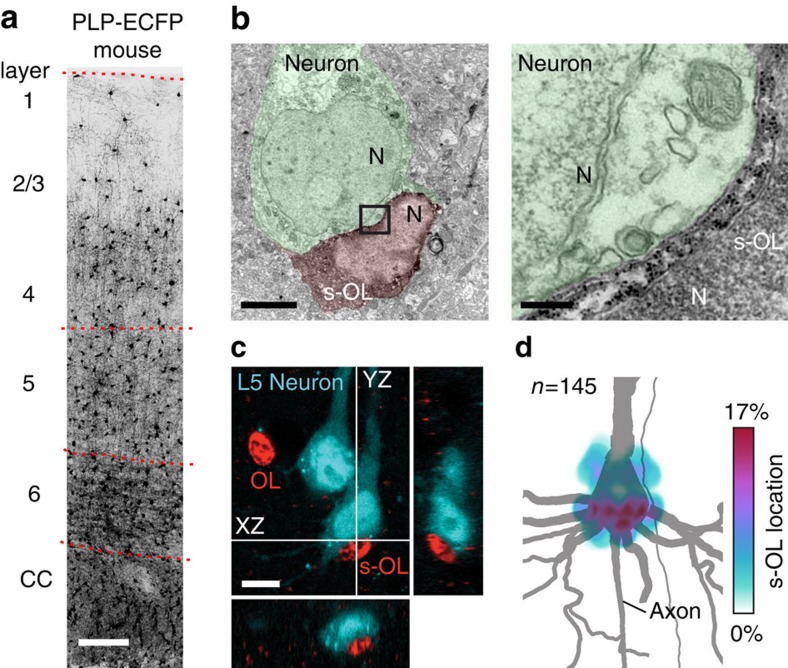
Identification and distribution of s-OLs around pyramidal neurons. (**a**) Overview image of oligodendrocytes in the somatosensory neocortex labelled for ECFP in the transgenic PLP-ECFP mouse. Scale bar, 150 μm. (**b**) Left: s-OL (red pseudo-colour) in a PLP-ECFP mouse neocortex exhibits positive immuno-gold labelling in the cell body. The host neuron is pseudocoloured in light green and nuclei are labelled with N. Scale bar, 3 μm. Right: higher magnification image of the box indicated in the left image, which shows a distinct and confined location of gold particle-labelled ECFP in the cell body (black dots). Scale bar, 200 nm. (**c**) Confocal image of labelled OLs in a PLP-ECFP mouse and two biocytin-filled L5 neurons and corresponding orthogonal views. The s-OL cell body is in direct contact with the neuronal soma. Processes of OLs are not visible because laser intensity was reduced to a minimum. Scale bar, 10 μm. Also, see Supplementary Movie 1. (**d**) Heatmap illustrating the location probability of 145 s-OLs in relation to the soma of an example L5 neuron (grey). s-OLs were positioned at multiple locations around the soma, but the highest percentages were found around the base of neurons close to the AIS and the basal dendrites. See also Supplementary Fig. 1.

To study s-OLs, we used a transgenic mouse line that expressed enhanced cyan fluorescent protein (ECFP) under control of a PLP promoter (PLP-ECFP, see Experimental Procedures; Fig. 1a) and focused on the neocortical layer 5 region. Electron microscopy identification of ECFP expressing s-OLs (Fig. 1b) revealed a characteristic very close apposition to neuronal cell membranes over long stretches with an interstitial space of 12.3±1.8 nm (*n*=4 cells). A close apposition was also seen in confocal analyses of neuron–s-OL pairs in which s-OLs were often found to fit in concavely shaped membrane regions of layer 5 neuron cell bodies and primary basal dendrites (Fig. 1c; Supplementary Movie 1). Estimates of the opposing membrane area yielded an average of 83±8 μm^2^ (range: 60–110 μm^2^, *n*=6). The initial description of s-OLs noted that these cells often locate to the base of the neuron soma^13^. To examine whether s-OLs have a preferred location around the pyramidal neuron cell body, we analysed tissue labelled for OLs and neurons. Confocal *z*-stack images had on average 46±5 OLs (24 regions of interest (ROIs), 3 animals and *n*=1,103 cells) with 34.4±1.4% being s-OLs (*n*=380). From all NeuN-labelled neurons, 18.4±2.0% were associated with a s-OL (Supplementary Fig. 1a,b). However, this likely underestimates the percentage of s-OLs at larger pyramidal neurons, since s-OLs preferentially reside near glutamatergic neurons^10^ and NeuN labels all neurons. For 145 randomly selected s-OLs, we plotted their position relative to the neuron soma. The data showed that s-OLs are positioned with higher probability around the soma base and basal dendrites (51.4%) than in the vicinity of the apical dendrite (21.5%) or between these subcellular locations (27.1%, *χ*^2^-test, *P*<0.0001, *n*=3 mice; Fig. 1d). Simultaneous labelling for OLs, β-IV-spectrin (an AIS marker) and NeuN revealed that the edge of s-OLs was on average 7.4±1.4 μm away from the onset of the AIS (*n*=26, Supplementary Fig. 1c).

### Identified layer 5 s-OLs myelinate axons

**Figure 2:**
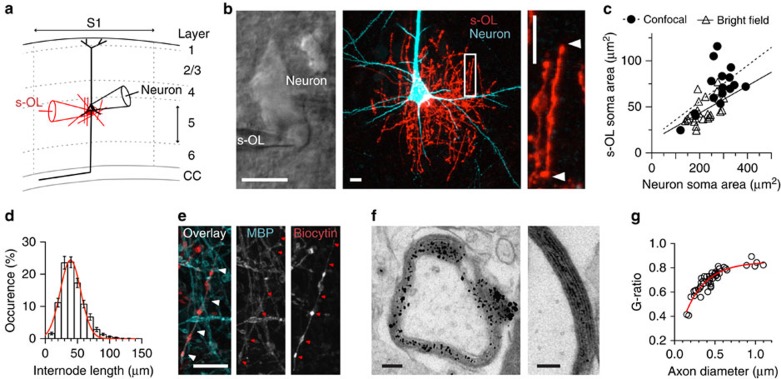
Experimental targeting of s-OLs reveals compact myelin and wrapping of surrounding axons. (**a**) Illustration of the experimental approach. s-OL (red) and layer 5 neuron (black) targeted with patch-clamp pipettes in mouse primary somatosensory neocortex. (**b**) Left: Bright-field image of a neuron–s-OL pair. Patch pipettes are visible. Middle, right: Confocal maximum *z*-projected image of a neuron–s-OL pair after intracellular loading with Alexa 488 (neuron) and Alexa 594 (s-OL) dyes. The white box is displayed at higher magnification on the right. White arrows indicate one internode. All scale bars, 10 μm. See Supplementary Movie 2. (**c**) Correlation of soma size between neurons and s-OLs as estimated from two separate data sets. Closed circles display data from maximum *z*-projected live-confocal scans and open triangles are measurements from bright-field images. Both data sets were fit by linear regressions (confocal scans: *n*=16, correlation coefficient *r*^2^=0.33, *P*=0.02; bright field: *n*=24, *r*^2^=0.29, *P*=0.007). (**d**) Distribution histogram of s-OL internode length measured from confocal live image *z*-stacks (*n*=32 cells). The data were fit by a Gaussian function (red line, *y*=24.36*e(−0.5*((*x*-39.30)/15.93)^2^)). Data presented are mean±s.e.m. (**e**) A s-OL was filled with biocytin and Alexa 594 during recordings and subsequently *post hoc* labelled for myelin basic protein (MBP). Internodal structures are positively co-labelled by MBP and biocytin (red and white arrowheads). Scale bar, 10 μm. (**f**) Left: electron microscopy image of an identified horseradish peroxidase (HRP)-filled transversal cut s-OL process shows multiple layers of myelin wrapped around an axon. Gold particles are visible as black dots in the outer tongue and all myelin layers, suggesting a direct cytoplasmic connection with the cell body. Right: a putatively different axon at higher magnification shows about seven gold-labelled compact myelin wraps. See also Supplementary Fig. 2. Scale bars, 100 nm. (**g**) The g-ratio measured from single identified s-OL axons (*n*=45) plotted as function of the axon diameter. s-OLs myelinate axons of variable diameter. The data were fitted with an exponential equation (*y*=−0.723 × e (−0.0038 × *x*)+0.85).

For electrophysiological recording and single-cell fluorescence characterization, we targeted putative s-OLs based on their position at the soma of medium- to large-sized layer 5 pyramidal neurons (Fig. 2a,b). s-OLs were recorded either individually (total *n*=70) or during s-OL–neuron paired recordings (total *n*=71 pairs). While various glia cell types can be in contact with neurons, OLs are most frequently in a satellite position^9^. In line, satellite glia cells of wild-type (WT) animals were in some cases astrocytes or OL precursor cells (combined ∼20%; Supplementary Fig. 1d). Quantification of the soma area from 16 neuron–s-OL pairs revealed that the soma size of s-OLs significantly and positively scaled with the host neuron cell body dimensions (Fig. 2c). Live-confocal scans showed that all s-OLs had internode-like structures oriented in vertical, horizontal and various diagonal directions, and resembled myelin based on the transmitted light images (Fig. 2b; Supplementary Movie 2; Supplementary Fig. 2a). s-OLs had on average 32±2 internodal-like branches with a mean length of 43.4±1.3 μm (*n*=32; Fig. 2d).

Myelination by s-OLs is unexpected as s-OLs are referred to as non-myelinating in the literature^10^,^12^,^14^,^15^. To further examine whether axon wrapping by s-OLs forms compact myelin, we pursued two independent approaches. First, biocytin- and Alexa 594-filled s-OLs were positively *post hoc* labelled for myelin basic protein (MBP; *n*=4; Fig. 2e). Second, to identify single s-OL internode–axon assemblies at the ultrastructural level, we filled s-OLs (*n*=5 cells) with horseradish peroxidase. Electron microscopy processing (Supplementary Fig. 2b) revealed that s-OLs made on average ∼4 myelin wraps (range: 3–7, *n*=12; Fig. 2f) around axons with diameters ranging between 0.2 and 1.1 μm (Fig. 2g; Supplementary Fig. 2c). The average g-ratio was 0.70±0.02 (*n*=45 axons, *n*=5 s-OLs; Fig. 2g). Notably, we found that s-OLs never myelinated the first internode of their host neuron. This is most likely explained by the length of the first internode of the layer 5 axon extending up to ∼110 μm from the soma edge, which is beyond the maximum expansion of the s-OL internodes (∼80 μm from the centre of the s-OL cell body, *n*=40). These data highlight that all s-OLs myelinate axons of varying small diameter with short internodal processes, classifying them as type 1 OLs^15^.

### s-OLs sense APs at high temporal resolution

**Figure 3:**
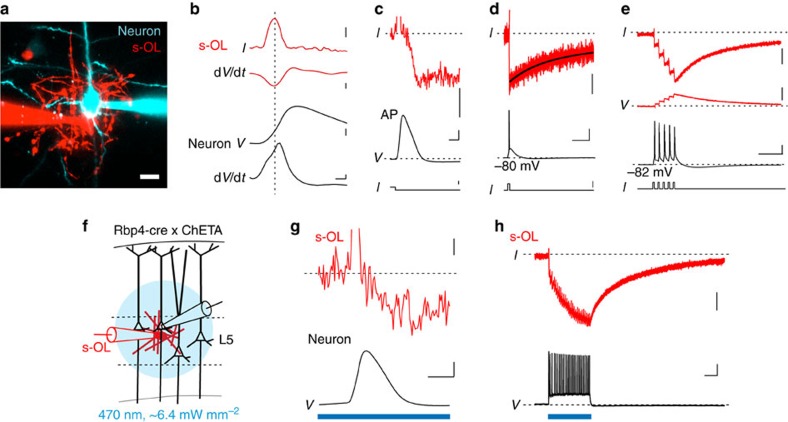
Action potentials evoke time-locked inward currents in s-OLs. (**a**) Confocal *z*-projected image of dual-whole-cell recording from L5 neuron–s-OL pair in the somatosensory neocortex. Scale bar, 20 μm. (**b**) The d*V*/d*t* of the AP aligns with the fast current transient in the s-OL, reflecting the capacitive charge of the s-OL membrane during the rising phase of the AP. In current clamp, the s-OL membrane voltage change is slow (d*V*/dt) compared with the neuronal AP. Note the d*V*/d*t* is from a different recording and filtered for display. The other traces are from the same cell as in **c** and **d**. Scale bars, (top to bottom) 10 pA, 1 V s^−1^, 30 mV (middle), 100 V s^−1^ and 0.1 ms. (**c**) AP repolarization temporally aligns with a rapid inward current in the s-OL. Average of 35 trials; scale bars, 5 pA, 30 mV, 500 μs and 0.7 nA. Capacitive current transient clipped for clarity. (**d**) The single AP evoked a rapid inward current in the s-OL (red trace, top) that decayed slowly (held at –91 mV; black fit, *τ*_1_=78 ms, *τ*_2_=451 ms). Traces displayed are the average of 25 trials. Scale bars, 2 pA, 30 mV, 50 ms and 1 nA. (**e**) Five APs evoked at 100 Hz potentiated the inward current amplitude in the s-OL (clamped to –88 mV, upper red trace). In current clamp (lower trace), the s-OL was depolarized by ∼500 μV. Traces are average of 26 trials. Scale bars, 10 pA, 500 μV, 30 mV and 50 ms, respectively. (**f**) Illustration of the targeted activation of layer 5 neurons by ChR2 while simultaneously recording from s-OL–neuron pairs. (**g**) Single light-evoked neuronal AP led to a capacitive transient (clipped) in the s-OL, followed by an inward current that activated during AP repolarization similar to electrical evoked AP. Scale bars, 2 pA, 30 mV and 0.5 ms. (**h**) The synchronised network activation of layer 5 neurons by ChR2 led to a large AP evoked inward current in a simultaneous recorded single s-OL. Scale bars, 20 pA (top), and 30 mV and 0.2 s (bottom).

Whole-cell recordings revealed that s-OLs have a resting membrane potential of on average –86 mV, a very low input resistance (*R*_N_) of ∼13 MΩ and a linear current–voltage relationship (Supplementary Table 1; Supplementary Fig. 3a–c). Given the close proximity to layer 5 somata and AIS, we tested in dual-whole-cell recordings (Fig. 3a) whether s-OLs respond to APs. In response to a single AP, s-OLs displayed a small and transient outward current (12.5±0.3 pA, *n*=23) temporally aligned with the capacitive charge during the AP depolarization (Fig. 3b). The capacitive transient was followed by an inward current with amplitude of on average –2.2±0.3 pA (*n*=7; Fig. 3c). Interestingly, the onset of this inward current aligned with the beginning of the repolarization of the AP and continued for several hundreds of milliseconds (Fig. 3d). Subthreshold current injections in neurons did not lead to detectable inward currents (*n*=6; Supplementary Fig. 3d). Since layer 5 neurons can generate APs at frequencies up to ∼300 Hz (ref. 19), we assessed the impact of a high-frequency train of APs and the response of s-OLs (Fig. 3e). Five APs with a frequency of 100 Hz resulted in an inward current of –15.9±1.8 pA in amplitude, cumulating with each AP and decaying double exponentially (*τ*_1_=39±4 ms, *τ*_2_=235±24 ms, *τ*_weighted_=74±9 ms, *n*=22). Due to the low input resistance in current-clamp configuration, five APs depolarized s-OLs by only 0.5±0.1 mV (*n*=5; Fig. 3e). The charge transferred per AP remained constant between 1 and 200 Hz (Kruskal–Wallis test, *P*=0.5, *n*=35; Supplementary Fig. 3e) and was on average 0.42±0.05 pC per AP. Varying the holding potential of s-OLs to positive voltages diminished, but did not abolish, the inward currents (Supplementary Fig. 3f). We next assessed the impact of a synchronously activated network of layer 5 neurons by photostimulating layer 5 neurons that expressed channelrhodopsin 2 (ChR2, ref. 20; Fig. 3f). Single APs of the host neuron were well discernible and similar to the response of electrically evoked inward currents (Fig. 3g). During long light pulses, inward currents had an average amplitude of –92±19 pA with a total charge of –136±36 pC that decayed double exponentially (*τ*_weighted_=377±113 ms, *n*=6; Fig. 3h). These results show that during *in vivo*-like synchronized activity of layer 5 pyramidal neurons, a substantial inward current is generated in s-OLs.

### AP-evoked currents are glial specific and Ba^2+^ sensitive

**Figure 4:**
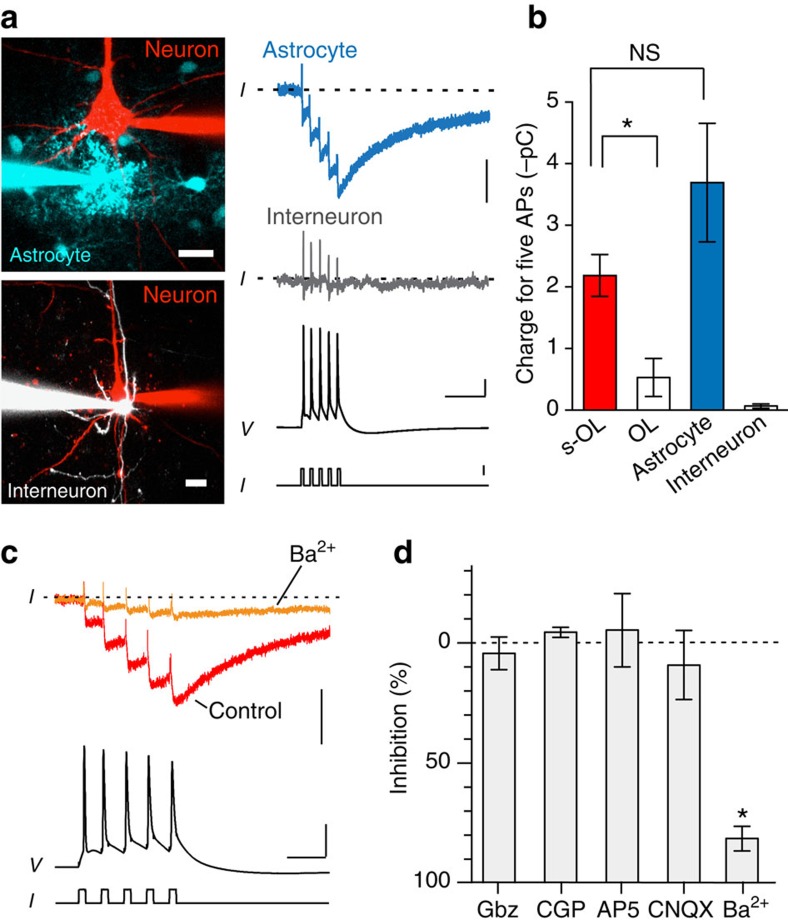
Inward currents are glial specific and Ba^2+^ sensitive. (**a**) Left: *Z*-projected confocal images of paired neuron–astrocyte and neuron–interneuron recordings. Scale bars, 20 μm. Right: traces from paired recordings show that astrocytes in satellite position show similar inward currents as s-OLs, but interneurons exhibit no inward currents; capacitive transients are still visible. The electrophysiology traces do not correspond to the image shown on the left. Scale bars, 10 pA (top and middle) and 30 mV, and 50 ms and 1 nA (bottom). (**b**) Summary data of the charge for five APs for s-OLs (*n*=22), oligodendrocytes not in satellite position (*n*=8), astrocytes (*n*=5) and interneurons (*n*=4) reveal that only glia in close proximity to the firing neuron exhibit a substantial inward current. Mann–Whitney test, *P*=0.0004 and *P*=0.129. Data are mean±s.e.m. (**c**) Left: extracellular application of 100 μM Ba^2+^ (orange) blocks the AP-induced inward current in s-OLs. Scale bars, 20 pA, 40 mV and 20 ms. (**d**) Population data of blocking experiments. Neither glutamatergic (50 μM D-AP5, *n*=3; 20 μM CNQX, *n*=2) nor GABAergic receptor blockers (5 μM gabazine, *n*=3; 50 μM CGP-35348, *n*=2) affected the inward current (for all Wilcoxon signed-rank test, *P*>0.18). In contrast, 100 μM Ba^2+^ led to an ∼80% reduction of the s-OL inward current (Wilcoxon signed-rank test, *n*=5, *P*=0.043). Data shown are mean±s.e.m.

We next asked whether these inward currents are specific for s-OLs. Paired recordings of pyramidal neurons and astrocytes in a satellite position on average 2±1 μm away (*n*=5, Mann–Whitney test, *P*=0.18, compared to s-OLs (*n*=22)) revealed inward currents of comparable amplitude (–23.26±7 pA, *n*=5, Mann–Whitney test, *P*=0.4) and decay kinetics (*τ*_weighted_=64±9 ms, *n*=5, Mann–Whitney, *P*=0.62, Fig. 4a,b). In contrast, AP-evoked inward currents were absent in interneurons that were in a satellite position to pyramidal neurons (–0.94±0.3 pA, *n*=4, Fig. 4a,b). OLs that were not in a satellite position to the recorded neuron (>10 μm membrane separation) had significantly smaller inward currents in response to APs (–2.1±1.1 pA, *n*=8, Mann–Whitney, *P*=0.0002, Fig. 4b) and were not detectable beyond an intercellular distance of 50 μm (*n*=4).

To identify the source of the inward current, we first applied blockers against ionotropic glutamate receptors, known to be expressed in adult OLs and OPCs^21^,^22^. However, the AP-evoked inward currents were unaffected by the NMDA (*N*-methyl-D-aspartate) receptor blocker D-AP5 (50 μM) or 20 μM of the kainate/AMPA (α-amino-3-hydroxy-5-methyl-4-isoxazole propionic acid) receptor blocker CNQX (ratio (ctrl/blocker) of 1.1±0.1 and 0.9±0.1, respectively, Wilcoxon signed-rank test, *P*=0.99, *n*=3; *P*=0.65, *n*=2; Fig. 4c). Similarly, blockers against GABA_A_ (γ-aminobutyric acid; 5 μM gabazine) and GABA_B_ (50 μM CGP-35348) receptors also did not affect the inward currents (0.96- and 1.04-fold change, respectively, Wilcoxon signed-rank test, *P*=0.65, *n*=3; *P*=0.18, *n*=2; Fig. 4c). Adult rat OLs express inwardly rectifying potassium (K^+^) channels at the cell body that are sensitive to barium (Ba^2+^)^23^. Following 100 μM Ba^2+^ application, the maximum amplitude of the AP-evoked inward current was reduced by 80.7±2.6% (Wilcoxon signed-rank test, *P*=0.043, *n*=5; Fig. 4c) and the decay time constant of the remaining current was significantly slowed (control: *τ*_weighted_=58±8 ms, Ba^2+^: *τ*_weighted_=505±148 ms, *n*=5, Wilcoxon signed-rank test, *P*=0.04).

On the basis of previous recordings of voltage-gated K^+^ currents in somatic outside-out patches from rat L5 neurons^24^ the K^+^ charge per AP was estimated to be 5.3 fC μm^−2^. The L5 soma width (14.4±0.3 μm, *n*=34) accounted for a somatic surface area of ∼707 μm^2^ (assuming a ball) and a single AP would thus generate a K^+^ charge of 3.7 pC. An individual s-OL, generating 0.42 pC charge per AP, thereby takes up ∼11% of the somatically extruded K^+^ ions. Assuming a cleft dimension (*δ*) of 20 nm (or 0.02 μm) between the neuron and the s-OL (Fig. 1b; Supplementary Movie 1), the numerical estimation of the K^+^ charge within the extracellular volume would be equivalent to 2.7 mM per AP (see Methods). Taken together, these data show that Ba^2+^-sensitive inward currents are specific for glia cells in direct contact or close proximity to the firing neuron, suggesting that they play a role in the activity-dependent K^+^ uptake.

### Kir4.1 channels are not mediating inward K^+^ currents

**Figure 5:**
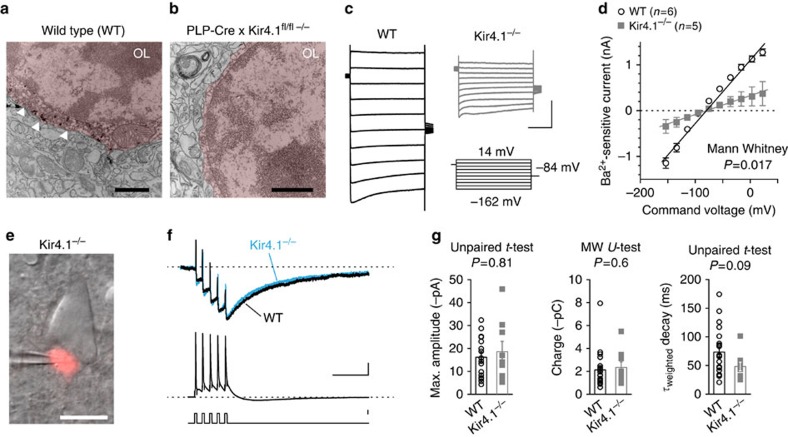
Oligodendrocytic Kir4.1 does not contribute to AP-evoked inward currents in s-OLs. (**a**,**b**) Electron microscopy images of WT and Kir4.1^−/−^ tissue immuno-gold labelled with an antibody against Kir4.1. In the WT tissue, gold particles are present (white arrowheads) at the soma that were absent in the Kir4.1^−/−^ OLs. Scale bars, 1 μm. (**c**) Ba^2+^-sensitive currents from WT (black) and Kir4.1^−/−^ (grey) s-OLs evoked by the displayed voltage steps. Scale bars, 0.5 nA and 0.1 s. (**d**) Summary of the experiments in **c** shown in an *I*–*V* curve. Kir4.1^−/−^ s-OLs have a reduced Ba^2+^-sensitive conductance. Data are mean±s.e.m. (**e**) Combined bright-field and tdTomato fluorescence example image of a Kir4.1^−/−^ s-OL–neuron pair in layer 5. Scale bar, 10 μm. (**f**) Recording of the Kir4.1^−/−^ s-OL–neuron pair shown in **e** overlaid with a recording from a WT experiment and the inward current evoked by five APs. s-OL currents were normalized to the maximum amplitude. Scale bars, 30 mV, 50 ms and 1 nA. (**g**) Summary data of the maximum amplitude, charge and weighted decay time constant reveal no differences of the action potential-evoked inward current properties between wild-type and Kir4.1^−/−^ s-OLs. Data are mean±s.e.m.

On the basis of our experiments (Figs 3 and 4) and a report of Ba^2+^-sensitive inward currents in OPCs and adult OLs^23^,^25^, we hypothesized that the potassium channel Kir4.1 is responsible for the observed inward current after AP firing. To test this hypothesis, we utilized a floxed Kir4.1 mouse^26^ in which we specifically removed Kir4.1 in adult OLs. After conditionally knocking out Kir4.1 (Kir4.1^−/−^) the deletion was confirmed by the absence of immuno-gold labelling at electron-dense OL cell bodies (Fig. 5a,b; Supplementary Fig. 4)^9^. In WT, gold particles were found in cell bodies but never in myelin sheaths, which is in agreement with previous published data^27^,^28^. To examine the consequence of Kir4.1 loss in OLs, we assessed the Ba^2+^-sensitive current in voltage clamp of Kir4.1^−/−^ and WT s-OLs (Fig. 5c). The Ba^2+^-sensitive conductance as estimated from steady-state I–V curves was significantly smaller in Kir4.1^−/−^ mice (4.1±2 nS, *n*=5) compared with WT s-OLs (13.1±1.9 nS, *n*=6, Mann–Whitney test, *P*=0.017; Fig. 5c,d; Supplementary Fig. 5a–c). Moreover, consistent with the sensitivity of Kir4.1 to intracellular spermine^29^, the Ba^2+^-sensitive current of wild-type s-OLs was modulated and reduced in outward rectification when spermine was added intracellularly (Supplementary Fig. 5d).

Next, we assessed in paired recordings from neurons and s-OLs the consequence of Kir4.1 knockout on K^+^ inward currents evoked by APs (Fig. 5e,f). Unexpectedly, an inward current was recorded in all pairs without a difference in their maximum amplitude (unpaired t-test, *P*=0.81), charge (Mann-Whitney test, *P*=0.6) or the weighted decay time constant (unpaired *t*-test, *P*=0.09) compared with WT (WT: *n*=16; Kir4.1^−/−^: *n*=9; Fig. 5g, WT data set same as Fig. 3). To further analyse the origin of the AP-mediated current in Kir4.1^−/−^ s-OLs, we applied Ba^2+^. Similar to WT s-OLs 100 μM Ba^2+^ reduced the AP-evoked current in Kir4.1^−/−^ s-OLs by 86±1 % (*n*=4 Mann Whitney test, *P*=0.11). In line with these data, s-OLs from Kir4.1^−/−^ animals appeared indistinguishable in their resting membrane properties compared with WT s-OLs (Supplementary Table 1). Thus, despite successful deletion of Kir4.1 in OLs and a reduction of Ba^2+^-sensitive current, the AP-dependent inward current and resting membrane properties are maintained. These data either suggest that other members of the Kir family or gap–junction coupled astrocytes contribute to the observed inward current.

### s-OLs connect to the astrocytic syncytium

**Figure 6:**
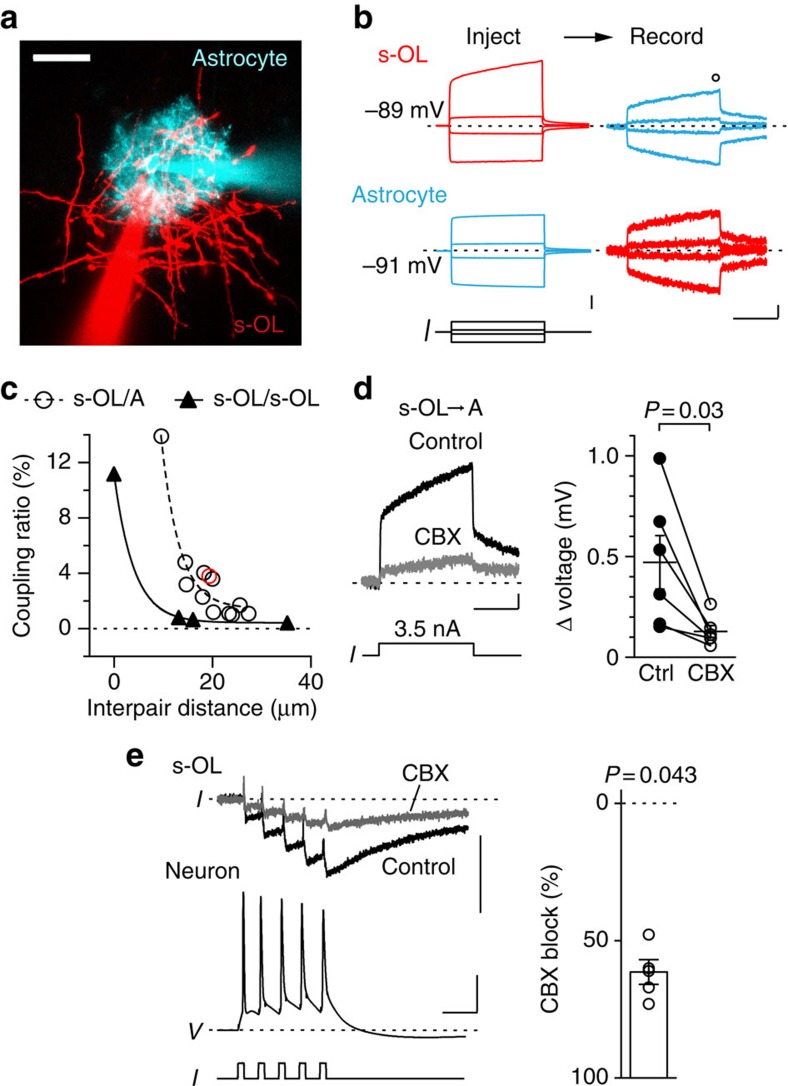
s-OLs are gap–junction coupled to other s-OLs and astrocytes. (**a**) Confocal *z*-projected image of a s-OL and astrocyte. Scale bar, 30 μm. (**b**) Gap–junction coupling between s-OL and astrocytes was estimated by bidirectional current injections into s-OLs or adjacent astrocytes and simultaneous recordings of the response in the corresponding cell. Traces are averages of 10 trials from pair in **a**. Open circle indicate the measurement region at the end of the pulse. Scale bars, (inject) 5 mV, (record) 200 μV and 50 ms, respectively. (**c**) Summary plot of the coupling ratio in the steady state as a function of the distance between recorded s-OL/astrocyte (*n*=12) and s-OL/s-OL (*n*=4) pairs. The open red circle indicates the experiment shown in **a** and **b**. Data were fitted by single exponential equations: s-OL/astrocyte: *y*=165.2 × e(−0.27 × *x*)+1.46 (dotted line); s-OL/s-OL: *y*=10.76 × e(−0.249 × x)+0.435 (black line). (**d**) Left: the gap–junction blocker carbenoxolone (CBX) reduced the cross gap–junctional current between s-OLs and astrocytes compared with control conditions. Scale bars, 0.1 mV and 50 ms. Right: summary data showing the inhibition of the gap–junction-mediated current (*n*=6, Wilcoxon signed-rank test, *P*=0.03). Data are mean±s.e.m. (**e**) Left: CBX reduced the AP-mediated inward current between neuron–s-OL pairs by 60%. Scale bars, 20 pA, 40 mV and 20 ms. Right: summary plot showing the reduction of the inward current in s-OLs in the presence of CBX in response to five APs (*n*=5). Data are mean±s.e.m.

Panglial dye coupling has been reported in acute grey and white matter slices^30^,^31^,^32^, electrical coupling between OLs and astrocytes has been shown to occur in explant cultures^33^ and gap–junctions are implicated in K^+^ buffering^34^. We therefore tested reciprocal electrical gap–junction coupling between the pairs of s-OLs and between s-OLs and neighbouring astrocytes (Fig. 6a,b). The average coupling ratio between s-OLs and astrocytes (*n*=12) was 3±1% and between OL–OL pairs (*n*=3) 0.6±0.1% (Mann–Whitney test, *P*=0.0044; Fig. 6c) when recorded from similar distances (Mann–Whitney test, *P*=0.82). Interestingly, the coupling was distance dependent with a strong attenuation at distances >20 μm (Fig. 6c). In addition, astrocytes were dye coupled with 9±1 other astrocytes within a domain (*n*=20). To test whether electrical coupling between s-OLs and astrocytes is mediated by gap–junctions, we bath applied 100 μM carbenoxolone (CBX). After wash in the voltage coupling between s-OLs and astrocytes was reduced by 67±6% (Wilcoxon signed-rank test, *P*=0.03, *n*=6; Fig. 6d). Finally, we tested the impact of CBX on the AP-evoked inward current in s-OLs. CBX caused a reduction in s-OL current amplitude by 58.1±4.4% (Wilcoxon signed-rank test, *P*=0.043 *n*=5; Fig. 6e) without changing decay kinetics (control: *τ*_weighted_=76±18 ms, CBX: *τ*_weighted_=136±46 ms, *n*=5; Wilcoxon signed-rank test, *P*=0.14). These results show that astrocytes and s-OLs in the vicinity of neuronal cell bodies are coupled by gap–junctions likely forming a syncytium to efficiently taking up K^+^ from the extracellular perisomatic region.

### Experimental and modelling predictions of potassium uptake

**Figure 7:**
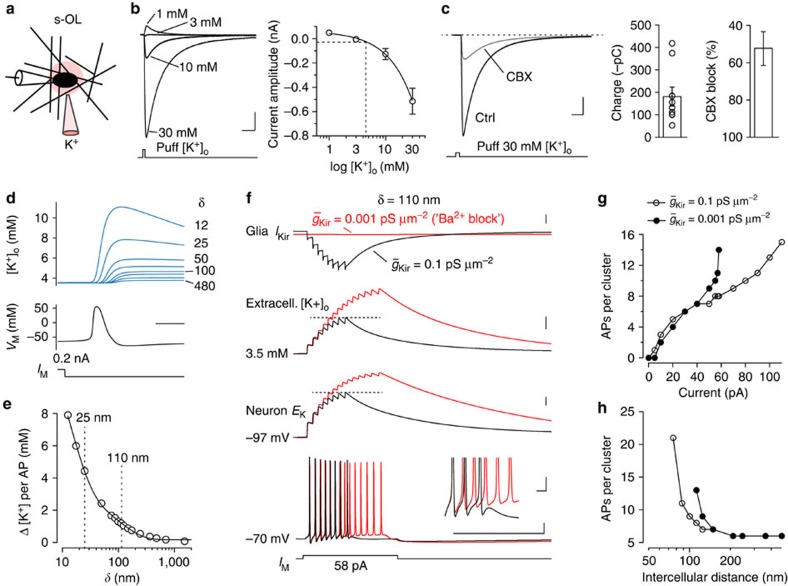
Glial Kir constraints high-frequency-dependent [K^+^]_o_ elevations. (**a**) Sketch of experimental [K^+^]_o_ application. (**b**) Left: average current responses to puffs of various [K^+^]_o_ at a constant holding voltage of −84 mV. Scale bars, 0.1 nA and 0.2 s. Right: Summary plot for all experiments fitted by a linear equation *y*=−19.2*x*+61.6 (1 mM: *n*=5; 3 mM: *n*=6; 10 mM: *n*=4; 30 mM: *n*=9). Dotted line indicates estimated [K^+^]_o_ from experimental data of five APs. Data presented as mean±s.e.m. (**c**) Response to local puff application of 30 mM [K^+^]_o_ and inhibition by 100 μM CBX. Scale bars, 0.1 nA and 0.2 s. Summary bar graphs for the total charge of the inward current during 30 mM [K^+^]_o_ and percentage of block after application of carbenoxolone (*n*=2). Data shown as mean±s.e.m. (**d**) Computer simulations of changes in [K^+^]_o_ at the soma evoked by a single AP, as a function of the intercellular distance (*δ*) between glia and neuron. Scale bar, 1 ms. (**e**) Summary plot of [K^+^]_o_ change in relation to the intercellular distances between neuron and glia to determine the modelling parameter (*δ*=110 nm). (**f**) Simulated Kir reduction can generate additional APs. Top to bottom: the Kir current in the glial compartment, extracellular [K^+^]_o_, neuronal *E*_K_ and neuronal membrane potential for high (black) and low (red) conductance densities of Kir. Reducing Kir increases the K^+^ reversal potential. Scale bars, (top to bottom) 20 μA cm^−2^, 2 mM, 5 mV, 100 ms and 20 mV. Inset: the onset of additional APs triggered by impaired Kir (red) shows the reduced fast AP afterhyperpolarization. Scale bars, 5 mV and 5 ms. (**g**) Input–output function of AP number within the high-frequency cluster versus current injection. Impact of glial Kir reduction only becomes apparent when APs occur at high frequencies (>∼170 Hz) with no impact on low-frequency AP generation (see Supplementary Fig. 6). (**h**) The positive feedback on AP firing by impaired Kir (closed dots) uptake becomes prominent in narrow interstitial spacing <100 nm (fixed current injection of 58 pA).

To examine how changes in [K^+^]_o_ relate to the s-OL inward currents, we applied K^+^ (1–30 mM) to the soma of s-OLs with pipettes closely positioned near the cell body (7.2±0.4 μm, *n*=21; Fig. 7a) and a holding potential of –84 mV. A [K^+^]_o_ application of 1 mM led to small transient outward currents (peak amplitude of 48.6±13.7 pA, *n*=5). However, when [K^+^]_o_ was higher than the control [K^+^]_o_ of 3 mM, inward currents increased up to a peak amplitude of on average –514±106 pA (*n*=9; Fig. 7b) at 30 mM [K^+^]_o_. By fitting these data with a linear regression (*R*^2^=0.99), we determined that the [K^+^]_o_ evoked peak currents reversed polarity at 3.2 mM. On the basis of the total s-OL surface area of ∼400 μm^2^ and an apposition area of ∼80 μm^2^ to the host neuron, we assumed that five times smaller current is generated at the s-OL soma during APs. In the adjusted linear regression (*y*=–3.8*x*+12.3), we substituted our peak amplitude current from five APs. Using the experimentally recorded range from –5 to –30 pA (Fig. 3e), we calculated a range of concentrations from 4.51 to 11.0 mM [K^+^]_o_. The average increase was up to 7.2 mM [K^+^]_o_ for five APs equivalent to ∼1 mM [K^+^]_o_ rise per single AP. To evaluate the contribution of astrocytes, we applied CBX while puffing 30 mM [K^+^]_o_. The inward current reduced amplitude and charge by 52±8% (*n*=2; Fig. 7c) indicating that gap–junction coupling to astrocytes may contribute for ∼50% of the inward current. These data suggest that the observed inward current is reflecting a participation of s-OLs in buffering [K^+^]_o_.

Next, we simulated the spatial K^+^ charge profile by a single AP in a published spatially accurate computational model of a L5 pyramidal neuron (Supplementary Fig. 6a). The model revealed that the AIS, soma and proximal dendrites are the main membrane sites contributing to the K^+^ efflux during the initiation and back propagation of a single AP strongly overlapping with the spatial arrangement of s-OLs. Given the critical role of the nearby AIS in determining the AP threshold in the axosomatic region^35^, we aimed to test whether local K^+^ buffering by the perisomatic syncytium influences neuronal excitability. Since Ba^2+^ is not specific for glial Kir, this excludes its use to distinguish the impact of glial and neuronal Kir. As an alternative, we first explored the impact of glial Kir-mediated K^+^ buffering on neuronal excitability using a published computational model with simplified neuronal morphology^36^,^37^. Varying the intercellular distance between neuron and glia in this model between 10 and 2,000 nm showed that AP-evoked [K^+^]_o_ increased between 8 and 0.1 mM, respectively (Fig. 7d). As expected, the AP-evoked [K^+^]_o_ increase was highly dependent on the extracellular volume; logarithmically reducing the intercellular distance between the neuron and glia (*δ*) increased [K^+^]_o_ (Fig. 7e) and a narrow spacing led to plateau depolarisations and seizure-like AP firing in the model cell (Supplementary Fig. 6b). An intercellular distance of *δ*=110 nm caused, however, a [K^+^]_o_ increase of 1.2 mM in line with our estimations for the perisomatic extracellular space. Plotting [K^+^]_o_ and *E*_K_ changes during AP firing revealed that reduction of glial Kir conductance density has an impact on the K^+^ driving force (Fig. 7f). With high glial Kir density (0.10 pS μm^−2^) [K^+^]_o_ accumulated to 8.5 mM. In contrast, a 100-fold reduction of Kir in the glial compartments led to a faster AP-dependent increase in interstitial [K^+^]_o_ up to a peak concentration of nearly ∼10 mM, and depolarized *E*_K_ by an additional ∼5 mV (Fig. 7f). As a result, the APs were associated with a reduced afterhyperpolarization, facilitating the generation of additional APs (Fig. 7f). In contrast, for low frequencies, the neuronal AP firing showed no differences between high or low Kir (Fig. 7g). With higher current injections (>58 pA), APs were generated at higher frequencies (>170 Hz) and reduction of Kir in the glial compartment led to more APs (Fig. 7f,g; Supplementary Fig. 6c). Indeed, when the extracellular volume was increased (*δ*>250 nm), the AP frequency reduced and glial Kir played no role in the initiation of APs (Fig. 7h).

Taken together, the modelling results suggest that placing glia close to the soma membrane causes two opposite changes. On the one hand, a narrower extracellular space increases neuronal excitability by amplifying activity-dependent [K^+^]_o_. On the other hand, the glia-specific K^+^ buffering limits [K^+^]_o_ and thereby prevents excessive AP generation. Moreover, glia-specific K^+^ buffering was in particular noticeable when APs occur at high frequency.

### Evidence for modulation of neuronal excitability by s-OLs

**Figure 8:**
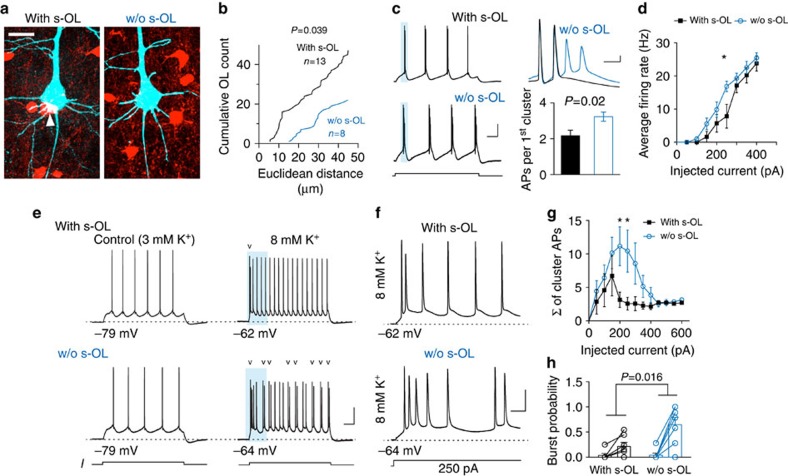
s-OLs limit high-frequency firing probability. (**a**) Maximum *z*-projected images of targeted L5 neurons (cyan) and surrounding oligodendrocytes (red). White arrowhead indicates s-OL. Scale bar, 20 μm. See also Supplementary Fig. 7a. (**b**) Distances of oligodendrocytes from the centre of neurons with or without s-OLs show differences in their spatial distances. (**c**) Left: trains of APs elicited by current injection steps for neurons with and without s-OL. Scale bars, 30 mV and 0.1 s. Top right: high temporal resolution of the first high-frequency AP cluster generated by L5 neurons with or without s-OL. Scale bar, 10 mV and 5 ms. Bottom right: summary data of the average APs per cluster for neurons with (*n*=6) and without s-OL (*n*=10, Mann–Whitney test, *P*=0.02). Data are mean±s.e.m. (**d**) Summary *F*–*I* plot of neurons with and without s-OL that generated high-frequency cluster APs. Differences in the average firing rate were observed at 250 pA (two-way ANOVA, *P*=0.0038). Data are mean±s.e.m. See also Supplementary Fig. 7b. (**e**) Left: APs evoked by steady-current injections from neurons with and without s-OL at normal extracellular K^+^ for selected regular firing neurons. Right: after increasing [K^+^]_o_ to 8 mM neurons depolarized and fired more APs with the same current injection. Arrowheads indicate cluster APs. Scale bar, 30 mV and 0.1 s. (**f**) Increased timescale of the traces in **d** in the presence of 8 mM [K^+^]_o_ shows more clustered APs in neurons without a s-OL. Scale bars, 30 mV and 25 ms. (**g**) Summary plot of the sum of cluster APs for neurons with and without s-OL indicate that the largest difference is observed for current steps between 200 and 300 pA (two-way ANOVA, *P*=0.0001, *n*=7 cells per group). Data are mean±s.e.m. (**h**) Summary plot displaying burst probability for neurons with and without s-OL in 3 and 8 mM [K^+^]_o_. at a current step of 200 pA. Data are single paired experiments and mean±s.e.m. Interaction significance was assessed with a two-way ANOVA-repeated measures.

To experimentally test whether the perisomatic syncytium around pyramidal neurons impacts neuronal firing, we took advantage of the fact that s-OLs are only found at ∼20% of L5 neurons (Supplementary Fig. 1b). Bright-field and fluorescence inspection in the PLP-ECFP mouse in combination with *post hoc* labelling of s-OLs confirmed their presence or absence (Fig. 8a; Supplementary Fig. 7a). Assessment of the local three-dimensional arrangement of OLs around these two neuron populations confirmed differences in proximity (with s-OL, *n*=13; without s-OL, *n*=8; Kolmogorov–Smirnoff test *P*=0.04; Fig. 8b). Comparative analysis of the intrinsic properties of L5 neurons with and without s-OL showed that the resting membrane potential of neurons lacking a s-OL (–76.4±0.5 mV, *n*=17) was on average 2.6 mV more depolarized (–79±0.4, *n*=51, unpaired *t*-test, *P*=0.002). Furthermore, the firing properties evoked by a steady depolarizing current step revealed that, while the large majority of neurons with s-OLs were regular firing (86%, 45 out of 51), L5 neurons without s-OL showed less regular firing (47%, 9 out of 19, Fisher's test, *P*=0.0007) and instead were more burst firing (53%, 10 out of 19). Firing behaviour of L5 neurons may be influenced by several factors including composition of intrinsic conductances, the dendritic morphology, as well as the axonal projection targets^19^,^38^. To isolate the role of extrinsic factors, we grouped L5 neurons according to their firing patterns (Fig. 8c). Analysis of the single APs from burst-firing L5 neurons revealed no difference in amplitude half-width, afterdepolarisation or the rate of rise during the AP onset (with s-OL *n*=6, w/o s-OL *n*=8, Supplementary Table 2). In addition, analysis of the *F*–*I* curve revealed neither a difference in the slope (*P*=0.7) nor the rheobase (*P*=0.11) of neurons that fired AP clusters. However, comparison of the first AP cluster revealed that neurons w/o s-OLs (*n*=10) generated significantly more APs per cluster compared with L5 neurons with s-OL (*n*=6, Mann–Whitney test, *P*=0.02; Fig. 8c). Furthermore, neurons without s-OL were on average firing at higher frequency at a current injection step of 250 pA (two-way analysis of variance (ANOVA), *P*=0.0038; Fig. 8d). In contrast, when the analysis was performed for neurons that were firing at regular low frequency, the presence of s-OLs had no influence on the firing properties (Supplementary Fig. 7b) and comparison of single AP properties revealed no differences (Supplementary Table 2), suggesting that the presence of s-OLs only affects repetitive spike generation.

Finally, the computational model predicted that glial Kir becomes critical when [K^+^]_o_ reaches higher concentrations, for example, during repetitive neuronal firing (Fig. 7g). Increased [K^+^]_o_ can then in turn facilitate the generation of additional APs due to depolarization of *E*_K_. To assess the impact of potassium buffering, we recorded from neurons with or w/o s-OL in baseline (3 mM) and elevated (8 mM) [K^+^]_o_ conditions (Fig. 8e). In 8 mM [K^+^]_o_, neurons in both the groups showed a strongly depolarized resting membrane potential (with s-OL: 13.5±1.2 mV, without s-OL 14.1±1.3 mV, *n*=7 cells per group, unpaired *t*-test *P*=0.7) and consequently the *F*–*I* curve showed a rheobase shift to lower thresholds (Supplementary Fig. 7c). In elevated [K^+^]_o_, neurons from both the groups generated high-frequency APs (Fig. 8e), which were more numerous in neurons w/o s-OL during current injections of 200 and 250 pA (two-way ANOVA, *P*=0.0001, *n*=7 cells per group; Fig. 8f,g). Subsequently, on the basis of these results, we calculated the burst probability for the 200 pA current step (Fig. 8h). Consistent with the model predictions of a large role of [K^+^]_o_ during bursts, the interaction analysis showed that the burst probability was more strongly increased in neurons w/o s-OL, further supporting our observation that the presence of s-OLs contributes to limit AP generation (repeated measures ANOVA, interaction *P*=0.016; Fig. 8h). In summary, these data suggest that the presence of s-OLs can be interpreted as a proxy for local differences in arrangement of the glial syncytium, which reduces the AP-dependent [K^+^]_o_ elevations and acts to limit the positive feedback on neuronal excitability.

## Discussion

Satellite oligodendrocytes are ubiquitously found throughout the cortex^10^,^12^,^17^ and hippocampus^39^ in rodents and humans^14^,^39^,^40^. While classified in the literature as non-myelinating similar to OPCs^10^,^12^,^14^,^15^, we present morphological, ultrastructural and molecular evidence that s-OLs abundantly myelinate axons indistinguishable from type I OLs (Fig. 2) reaffirming their original classification by del Río Hortega^13^.

What is the functional role of the close apposition of the outer membranes of OLs and neurons? Previous work suggested that s-OLs may provide metabolic support for neurons^10^, protect neurons against apoptosis^17^ or remyelinate axons following injury^12^. Specific arrangements between neurons and glia cells in satellite position, such as the cerebellar Bergmann glia, dynamically regulate extracellular K^+^, thereby ensuring firing bistability of the Purkinje cell^7^,^41^. The perisomatic domain of pyramidal layer 5 neurons generates substantial outward K^+^ current when APs back propagate from the axon into the soma and dendritic tree, and activate fast voltage-gated K^+^ channels^24^. It is well established that buffering of [K^+^]_o_ and water in the brain is a complex process controlled by passive diffusion, K^+^ uptake through Na^+^/K^+^ pumps and Kir channels as well as spatial K^+^ redistribution via electric coupling of glial cells^34^,^42^,^43^,^44^,^45^. AP-evoked K^+^ release into the extracellular space may be most efficiently controlled when glial syncytia are organized perisomatically and in close contact with the neuronal membrane. Consistent with these features we found that AP-evoked inward currents in s-OLs were highly sensitive to Ba^2+^ a Kir channel blocker and partially gap–junction mediated. Importantly, these currents were not different from perisomatic astrocytes that are widely implicated in K^+^ uptake (Figs 3, 4, 5, 6; refs 6, 34, 41, 44). Based on these results it may be postulated that s-OLs participate in regulation of extracellular K^+^.

Given the electrical coupling of s-OLs to an astrocyte syncytium what is the role of a satellite OL? The conditional OL-specific knockout of Kir4.1 was characterized by impaired Ba^2+^-sensitive currents strongly supporting the finding that Kir4.1 is at the membrane in mature OLs (Fig. 4)^28^,^46^. Unexpectedly, OL Kir4.1 was not required for AP-evoked inward current and setting the resting membrane properties. These results contrast with deletion or mutation of Kir4.1 in astrocytes causing impaired K^+^ siphoning, myelin defects and epilepsy^26^,^47^,^48^,^49^. There are several possibilities that can account for the lack of detectable impact on K^+^ uptake in Kir4.1^−/−^ s-OLs. First, since mature OLs are connected to the astrocyte syncytium (Fig. 5)^30^,^31^,^32^,^50^, the electrical coupling maintains *V*_M_ within a glial syncytia even when [K^+^]_o_ varies. Recent work showed that an ∼30% reduction in the number of astrocytes maintains the isopotentiality of a syncytium ensuring a constant driving force for K^+^ uptake^51^. Indeed, the disruption of OL gap–junctions Cx32 and Cx47 have been implicated in deficits of potassium and water siphoning, and myelination defects^34^. Given the dominant role of electrical coupling Kir4.1 activity of a single s-OL may thus be masked by the syncytium. Second, the lack of impact of OL-specific Kir4.1 knockout on the inward current may reflect a differential molecular composition of Kir expression in OLs and astrocytes. OLs express homotetramers of Kir4.1 (ref. 28), a feature which is shared with the endfeet of retinal astrocytes around capillaries^52^. Astrocytes, on the other hand, express heterotetrameric complexes of Kir4.1/Kir5.1 characterized by an approximately fourfold larger single-channel conductance compared with Kir4.1 homotetramers^28^,^53^,^54^. Third, it cannot be excluded that OL-specific uptake of K^+^ is complemented by Kir2.1, although this channel is expressed at lower levels compared with Kir4.1 (refs 28, 55). Furthermore, a dominant role of astrocytic Kir4.1 in a OL–astrocyte syncytium is supported by the Kir4.1 knockout studies showing not only deficits in potassium buffering and glutamate uptake but also white matter vacuolization^26^,^48^,^49^, which could be a consequence of elevated [K^+^]_o_ leading to myelin degeneration^56^. In line, inward currents of white matter OLs are predominantly carried by K^+^ (ref. 56) supporting an important role for OL physiology. On the basis of these and our present findings, further experiments are required to examine the properties of perisomatic astrocytes and OLs in local AP-evoked K^+^ uptake using the specific conditional astrocytic knockout of Kir4.1 or directly assessing [K^+^]_i_ by potassium-sensitive dye imaging^56^.

The Nernst equation predicts that depolarization of *E*_K_ reduces the driving force for K^+^, which causes membrane depolarization and increased neuronal excitability. Our computational simulations demonstrated that due to the fast temporal dynamics of Kir-mediated uptake, conducting logarithmically with [K^+^]_o_ (refs 29, 57), *E*_K_ is mostly influenced during high-frequency APs (Fig. 7). In good agreement with these predictions, the presence of s-OLs was associated with neuronal excitability changes selectively during high-frequency burst firing and as a function of the baseline [K^+^]_o_. These findings may suggest that s-OLs have a role in anatomically organizing the perisomatic glial syncytium. This hypothesis remains to be addressed in future studies using the conditional knockout of Kir4.1 in astrocytes or directly visualizing the entire perisomatic glial syncytium. Our results predict that perisomatic syncytia increase the diversity of populations of pyramidal neurons in the neocortex. Since high-frequency burst firing of neocortical pyramidal neurons occurs *in vivo* during active whisking behaviours and during coincidence detection^58^,^59^, perisomatic syncytia potentially may contribute to modalities of sensory integration.

In summary, the present work shows that satellite OLs are typical type I OLs and an integral component of an electrically coupled glial syncytium-mediating K^+^ buffering in the perisomatic domain. The results add to an emerging view that by regulating extracellular K^+^ or Ca^2+^ concentrations glial cells locally shape intrinsic excitability and rhythms in neural circuits^4^,^5^,^6^,^44^. Neuron–OL–astrocyte interactions may thus not only be critical for driving axonal myelination but also to regulate neuronal excitability on a millisecond timescale.

## Methods

### Animals

All procedures involving experimental animals were in agreement with European Union (EU) and national law, complied with guidelines of the Royal Netherlands Academy of Arts and Sciences (KNAW) and were approved by the local animal ethics committee (DEC). Male C57Bl6/J mice were obtained from Harlan or bred in-house. Mice expressing ECFP under the OL-specific PLP promoter (line Q—also PCFQ; here referred to as PLP-ECFP) were obtained from Frank Kirchhoff (University of Saarland) and this line was generated similarly to mice described earlier and expression was specific for OLs^60^. For breeding, PLP-ECFP-positive animals were crossed with C57Bl6/J wild-type mice. Genotyping was performed with a standard PCR protocol (annealing at 60 °C, extension at 70 °C, denaturation at 94 °C and 35 cycles) and the primers forward: 5′- ATGCGTACCTGACTTTCTCCTTCT -3′ and reverse: 5′- ACTGGGTGCTCAGGTACTGGTTGT -3′, which amplified a 750-bp fragment. For photostimulation experiments, Rbp4-cre_KL100Gsat/Mmucd mice (#031125-UCD, MMRRC) were crossed with R26-CAG-LSL-2XChETA-tdTomato mice (17455, Jackson Laboratory) to obtain layer 5 specific expression^20^ of the ChR2 variant ChETA. Oligodendrocytic-specific knockout of Kir4.1 was achieved by crossing Kir4.1^fl/fl^ mice^26^ with PLP-cre/ERT mice (#5975, Jackson Laboratory) and the fluorescent reporter mouse strain B6;129S6-*Gt(ROSA)26Sor*^*tm14(CAG−tdTomato)Hze*^/J (#7908, Jackson Laboratory). Primers used for genotyping were as described^26^ or as specified by the commercial sources. To induce Cre expression PLP-cre x Kir4.1^fl/fl^ mice were intraperitoneal injected with 75 mg kg^−1^ tamoxifen (Sigma-Aldrich) dissolved in corn oil for 4 or 5 consecutive days between postnatal days 35–61. Experiments were performed 22±7 days (*n*=9) after start of the injection. Kir4.1^−/−^ mice were monitored and did not exhibit abnormal weight changes or developed neurological symptoms as reported for conditional astrocytic or conventional Kir4.1 knockout mice^26^,^48^. All mice were housed in the institutional animal facilities on a 12-h light/dark cycle with light from 7:00 to 19:00 hours and food (Teklad global diet #2918, Harlan) and water *ad libitum*. All experiments were performed with 3–15-week-old (mean 53±4 days) mice of both sexes (90% male and 10% female) with a weight of 22.6±1.2 g.

### Chemicals

Salts for electrophysiology, gabazine, carbenoxolone and biocytin (all Sigma-Aldrich), DAP-5, gabazine, CGP-35348 and CNQX (Tocris) were dissolved in double-distilled water and when feasible stocks of blockers were stored in aliquots at –20 °C. Blockers were bath applied unless otherwise noted.

### Electrophysiology

For electrophysiological recordings, mice were deeply anaesthetized with 3% isoflurane, decapitated, the brain was quickly removed and placed in ice-cold carbogenated (95% O_2_, 5% CO_2_) cutting solution comprised of (in mM) the following: 125 NaCl, 25 NaHCO_3_, 1.25 NaH_2_PO_4_, 3 KCl, 25 glucose, 1 CaCl_2_, 6 MgCl_2_ and 1 kynurenic acid. Parasagittal slices of the neocortex were cut at 300-μm thickness (VT1200S, Leica Microsystems, Germany) and incubated at 35 °C for 35 min before storing in the same solution at room temperature. Slices were placed in a submerged chamber under an upright microscope (Olympus Nederland BV, Zoeterwoude, The Netherlands) and perfused with heated (33±2 °C) carbogenated artificial cerebrospinal fluid (ACSF) consisting of (in mM) the following: 125 NaCl, 25 NaHCO_3_, 1.25 NaH_2_PO_4_, 3 KCl, 25 glucose, 2 CaCl_2_ and 1 MgCl_2_ for recordings. Intracellular solutions for neuronal recordings consisted of (in mM) the following: 130 K-gluconate, 10 KCl, 10 HEPES, 4 Mg-ATP, 0.3 Na_2_-GTP, 10 Na_2_-phosphocreatine and pH set to 7.25 (∼280 mOsm). For OLs, we routinely used (in mM) the following: 130 K-gluconate, 4 NaCl, 0.5 CaCl_2_, 10 EGTA, 10 HEPES, 4 Mg-ATP, 0.3 Na_2_-GTP, pH set to 7.3 with KOH (∼280 mOsm). In a subset of cells, we additionally included 5 mg ml^−1^ biocytin. Borosilicate glass pipettes filled with intracellular solution had open pipette resistances of 5–7 MΩ. For identification and morphological analyses, intracellular solutions were supplemented with 200 μM Alexa 488 or 75–200 μM Alexa 594 (Life Technologies, USA) dissolved in intracellular solution. This experimental approach allowed identification and distinction of targeted cells, and we could subsequently discern s-OLs, astrocytes and OPCs using live-confocal scans. s-OLs showed multiple internodes of varying lengths, which was in contrast to astrocytes that had a bushy and dense appearance, lacked internodes and were dye coupled with other cells. OPCs exhibited multiple processes of shorter length than s-OLs, were not dye coupled, and lacked bushy processes. The liquid junction potential was corrected for and was calculated (pCLAMP10, Molecular Devices) to be –15 mV for the neuronal and –14 mV for the OL intracellular solution. For some experiments, we recorded from OLs using the same intracellular solution as for neurons. The different intracellular solutions did not lead to differences in intrinsic membrane properties of OLs (ANOVA: resting *V*_M_ (*P*=0.15); *R*_N_ (*P*=0.16); capacitance (*P*=0.68)) and data were subsequently pooled.

All electrophysiology experiments were controlled and recorded by Axograph X software (v1.3.5, AxoGraph Scientific, Sydney, Australia) and digitized by an AD board (ITC-18, HEKA Elektronik, Lambrecht, Germany). For voltage-clamp recordings, an Axopatch 200B amplifier (Molecular Devices, Sunnyvale, CA, USA) was used. All signals were filtered with the built-in 5 or 10 kHz Bessel filter and sampled between 20 and 50 kHz. Series resistance was on average 17.6±0.7 MΩ (*n*=100) and not compensated for in voltage-clamp recordings. From experiments in which we applied series resistance compensation and prediction by on average 84±2% and ∼75–100% (*n*=5), we observed an increase of current amplitude by 60±16% (*n*=5) but no alterations of the current kinetics (*n*=3). In current-clamp recordings of neurons and/or glia cells, bridge balance and capacitance compensation were fully applied (BVC 700A amplifier, Dagan Corporation, Minneapolis, MN, USA). Some current-clamp recordings of the OLs were made with an Axopatch 200B amplifier using the built-in I-clamp fast mode with bridge balance and capacitance fully compensated. Single APs were evoked by short step current pulses of 3 or 5 ms adjusted in amplitude to initiate after the stimulus end. Local puffing of solutions was performed with normal patch pipettes connected to a pressure application system (Picospritzer III, Intracel, Herts, UK) with pressure intensity adjusted to the duration of the pulse (20 ms) and location of the puff (0.1–0.3 bar). The application of different potassium concentrations (1, 3, 10 and 30 mM) was achieved by adjusting the sodium/potassium ratio of a HEPES-buffered standard ACSF accordingly. For example, for a 10 mM K^+^ solution, it was composed of (in mM) the following: 118 NaCl, 10 KCl, 10 HEPES, 2 CaCl_2_, 1 MgCl_2_, 25 glucose and pH set to 7.4 with NaOH. In experiments that involved application of 8 mM K^+^, we added 5 mM KCl to the standard ACSF. With an intracellular [K^+^]_i_ of 140 mM, this elevation of [K^+^]_o_ depolarized *E*_k_ from –101.4 to –75.5 mV (33 °C). The calculated change of *E*_Cl_ was only about –1 mV. When 8 mM K^+^ was bath-applied experiments were interleaved between neurons with and without s-OL. Experimenter was not blinded for experimental conditions or genotypes.

### Analysis of electrophysiological data

Analyses of electrophysiology recordings were performed with Axograph X. *R*_N_ was determined by fitting the linear range of voltage responses to current injections (±50 pA for L5 neurons and up to ±400 pA for OLs) around the resting membrane potential. The resting membrane potential was determined from traces with zero-current injection or alternatively read from the built-in display after breaking into whole-cell mode (Axopatch 200B). Resting conductance (*G*) was calculated from voltage-clamp recordings as *G*=Δ*I*/*V* were *V* was a 10 mV hyperpolarizing voltage step from –70 mV and Δ*I* the respective current difference. AP parameters were determined from single-evoked APs or steady-current injections as indicated. The threshold was determined when the slope of the voltage reached >50 V s^−1^ (refs 35, 61). Single APs were only analysed when they initiated with a delay after the end of the current pulse.

Gap–junction coupling of cells was tested by injecting current in one cell and recording the voltage response in both cells; current injections to assess coupling were performed reciprocally. For the analysis, current responses of 10 trials were averaged and the voltage was measured at the end of each current injection step for each cell. The coupling ratio (*V*_Receiving_ cell /*V*_Injected_ cell) was calculated for each current step and averaged for all positive and negative current injections. Subsequently, an average coupling ratio was calculated for each cell pair. The slopes of the *F*–*I* curves were estimated from linear fits to either all available data points (regular firing neurons) or to the first data points that were linear (burst-firing neurons). The rheobase was determined as the *x* interception of the linear fit when *y*=0. Regular and burst-firing neurons were grouped by their ability to generate repetitive APs at high frequencies^19^. Neurons with a short burst of two or more APs ⩾90 Hz (cluster) at the onset of the current injection were defined as high-frequency firing neurons and neurons with >10 Hz interspike frequencies as regular firing neurons.

### Photoactivation of layer 5 neurons

For photostimulation of the ChR2 variant ChETA in layer 5 neurons, we used a blue light-emitting diode (LED; peak at 470 nm, Thorlabs Inc, Newton NJ, USA) mounted into the light path of the microscope. For the experimental conditions, the LED output power was directly measured in the optical path (∼1.8 mW) with an optical power metre (Newport, Irvine, CA, USA). With this LED intensity, a short light pulse of 3 ms was sufficient to evoke single APs in layer 5 neurons. To control the area of illumination, a field stop was coupled into the excitation light path.

### Live imaging

During electrophysiological recordings, live fluorescence *z*-stacks were obtained with an upright microscope equipped with a confocal scanning unit (Olympus BX61/FV1000, Olympus) and laser lines for 488 and 594 nm (multi-line argon laser (Showa Optronics, Tokyo, Japan); helium–neon laser (Melles Griot, Carlsbad, CA, USA)). Images were acquired with either × 40 numerical aperture (NA) 0.8, × 60 NA 1.0 or × 60 NA 1.1 Olympus water immersion objectives in *z*-step sizes of 1 μm (Fluoview software, Olympus).

### Immunohistochemistry and image analysis

For *post hoc* labelling of intracellular dye-filled cells slices were fixed in 4% paraformaldehyde (PFA) for 10–20 min before further processing. We used antibodies against MBP (rabbit polyclonal anti-MBP, #AB980, Millipore or monoclonal mouse anti-MBP, #SMI-99P, Covance both 1:250), GFP (ab65556, 1:1,500, Abcam or 75-132, 1:100, Neuromab), Kir4.1 (rabbit polyclonal #APC-035, 1:300, Alomone, Israel), NeuN (polyclonal guinea-pig, 1:1,000, #ABN90P, Millipore) and NG2 (polyclonal rabbit, #AB5320, 1:250, Millipore). Corresponding secondary antibodies were conjugated with Alexa, 488, 555, 594 or 633 (1:500, all Life Technologies) and chosen as suitable. Immunohistochemical labelling was performed as described below. Kir4.1 immunofluorescence labelling was performed on tissue that was fixed for 20 min in 4% PFA and subsequently processed identical as described below.

For quantifying the distribution of OLs in neocortical layer 5, we immersion-fixed the brains of three mice (C57BL/6, ∼75 days) in 4% PFA for 4 h and cut slices of 40-μm thickness on a vibratome (VT1000S, Leica Microsystems, Germany). Sections were labelled with a polyclonal MBP antibody (AB980) of which some isoforms are known to be present in the cytoplasmic domain of OLs^62^ and a NeuN antibody (ABN90P), a pan-neuronal marker, in the presence of 1% Triton-X 100 at 4 °C overnight after blocking for 2 h at room temperature in the same solution without primary antibodies. Secondary antibodies were incubated for 2 h at room temperature and we used a goat anti guinea-pig-Alexa488 and goat anti rabbit-Alexa555 (Life Technologies). Subsequently, the sections were coversliped with Vectashield with 4,6-diamidino-2-phenylindole (DAPI; Vector Labs). For each animal, eight ROIs from four randomly selected slices in the layer 5 of the somatosensory neocortex were selected and 40 μm *z*-stacks were scanned on an inverted confocal microscope with a × 40 PlanApo objective 1.3 NA (Leica SP5, Leica Microsystems) zoom set to 2 and a *z*-step size of 1 μm (total volume ∼1,501,563 μm^3^). For the distribution analysis of s-OLs, we first analysed the distribution of all NeuN-positive cells and all cell nuclei that were identified by DAPI. NeuN and DAPI images were *z*-projected, thresholded and neurons, respectively, nuclei manually separated using the line tool. An automated analysis to detect the number of neurons and nuclei was performed by the analyse particles tool with ‘exclude on edges' activated (FIJI). Detection of oligodendrocytic cell bodies was performed manually for each *z*-stack. In Adobe Illustrator (Adobe), relative positions of 145 cell bodies were charted to a two-dimensional soma outline obtained from a mouse layer 5 neuron and further processed to create a distribution heatmap^63^. Each OL position was represented as a single point image (brush 5 point and line thickness 4 point), which approximately matched the relative proportions of s-OL cell bodies at L5 neurons. Each position was exported as individual black and white TIFF file, imported into FIJI and converted to binary. Thus, each OL containing pixel was defined as 100%. We then calculated the sum of all images and divided it by the maximum pixel value to obtain the percentage of each pixel to contain a s-OL. A Gaussian blur filter with radius set to 10 was applied and the look-up table (LUT) set. No further adjustment was applied to the images. The two-dimensional cell body area was estimated by outlining the soma area with the polygon selection tool (FIJI)^64^ from distance calibrated average *z*-projected confocal stacks acquired from acute brain slices. All other measurements were performed on either bright-field or confocal images acquired from live tissue. Length measurements were performed on calibrated bright-field or confocal images. The distance between two cells was measured from cell-to-cell border. OL surface area was estimated based on an ellipsoid shape and radii of 5.6, 3.7 and 8.2 μm (average values for *n*=18 cells). Internodal length was measured from s-OLs that were individually filled with fluorescence dyes and we could follow single internodes throughout a *z*-stack. We assumed that the internodal length corresponded to the visible internode and measured the length from beginning to end using the segmented line tool. Distance differences between cells in *xyz* coordinates were converted to Euclidean distance by 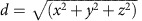.

### Electron microscopy

To obtain electron microscopy images from single s-OLs, we first identified s-OLs in acute slices of PLP-ECFP mice and filled them with 4–5% horseradish peroxidase type VI (Sigma-Aldrich) for >40 min during whole-cell recording. After pipette removal slices containing a single OL were fixed for ∼17 min in freshly prepared 5% glutaraldehyde in Na-cacodylate (0.1 M, pH 7.4), and then washed in Na-cacodylate buffer and cryoprotected in 25% sucrose. After saturation slices were embedded in an aluminium boat, frozen on dry ice and resectioned at 40 μm with a freezing microtome. For the peroxidase reaction, thin sections were incubated in a Tris-HCl buffered diaminobenzidine solution containing 0.03% H_2_O_2_. The diaminobenzidine reaction product was subsequently intensified by a gold-substituted silver peroxidase method^65^. Sections were then rinsed in sodium cacodylate buffer (0.1 M, pH 7.4) and post fixed for 20 min in 1% OsO_4_ supplemented with 1% ferricyanide in cacodylate buffer. Sections were washed, dehydrated and embedded in epoxy resin. Ultrathin sections (∼60 nm) perpendicular to the cortex were made (Ultracut E, Reichert-Jung/Leica), mounted on electron microscopy grids and contrasted with solutions of lead citrate and 0.5% uranyl acetate (Ultrastain 1 and 2, Laurylab, Brindas, France). Sections were observed with a transmission electron microscope (Tecnai 12, FEI Europe, Eindhoven, Netherlands) and digital images were acquired as TIFF files with a mega view 3 camera (Soft Imaging System/Olympus). The g-ratio for single labelled axons (longitudinally or transversally cut) was calculated from calibrated electron microscopy images as the diameter of the axon divided by the total diameter of the axon including the myelin sheath using FIJI. Major dense lines were counted from images that showed distinct single myelin layers. For antibody labelling of neocortical tissue, freshly prepared 300-μm slices were fixed at room temperature for 10 min in 4% PFA (0.1 M PB, pH 6.5) and then in 4% PFA (0.1 M NaHCO_3_ buffered, pH 10.4 for 10 min)^66^. After rinsing in PB (0.1 M, pH 7.4), the tissue was cryoprotected, frozen and 40-μm cryosections were prepared. Sections were then incubated with a rabbit polyclonal antibody against Kir4.1 in PB (1:300, Alomone) for 48 h at 4 °C. After rinsing, the sections were incubated with a BrightVision Poly-HRP-Goat-Anti-rabbit IgG (Immunologic, Duiven, The Netherlands). Tissues from wild-type and Kir4.1^−/−^ animals were processed in parallel. In Kir4.1^−/−^ sections, surrounding astrocytic processes exhibited gold particles, indicating successful labelling of the sections (Supplementary Fig. 4a–d). All other steps were performed as described above.

### Potassium concentration estimation in the soma s-OL cleft

To estimate the extracellular K^+^ concentration [K^+^]_o_ in the cleft region between the L5 soma and s-OL, we used an analytical solution as a function of cleft height (*δ*). Our previous outside-out recordings from rat layer 5 pyramidal neuron somata showed that the K^+^ charge per AP is ∼5.3 fC μm^−2^ (ref. 24). The measured spacing between a neuron and s-OL is probably affected by aldehyde fixation^67^ and therefore we use a published value of ∼20 nm (ref. 9). Assuming the cleft height (*δ*) is 20 nm (or 0.020 μm), the K^+^ charge in the extracellular volume may reach 260.5 fC μm^−3^ (260.5 × 10^−15^ C μm^−3^). The amount of charge divided by Faraday's constant (96485.336521 C mol^−1^) reveals a concentration corresponding to 2.699 × 10^−18^ mol μm^−3^ or ∼2.7 mol m^−3^ (mM). This upper boundary estimation is within range of previous calculations for synaptic clefts in the frog neuromuscular junction, yielding Δ[K^+^]_o_ of 2.1 mM per AP^61^. Taking into account the normal extracellular potassium concentration (3 mM) single APs from the layer 5 soma are expected to cause a peak [K^+^]_o_ within the cleft between the soma and s-OL of 5.7 mM. For simplicity, we ignored the lateral and radial K^+^ diffusion (*D*=1.96 × 10^−9^ m^2^ s^−1^) and assumed that all potassium ions will be instantaneously released in the cleft region.

### Electrodiffusion NEURON model

To predict the impact of glial Kir4.1 on neuronal AP firing, we used a previous published computational model^36^,^37^ available at ModelDB (accession ID: 113446). Simulations were performed using the NEURON platform (v7.3). The model consists of a simplified neuron with a somatic compartment and connected with several dendritic compartments containing voltage-gated sodium, potassium, calcium and calcium-activated potassium conductances, as published in detail previously^36^,^37^. The interstitial space in this model is uniform and implemented as a compartment in which ionic diffusion and buffering takes place as the interstitial space is itself connected with compartments representing glial, segmented with the same spatial resolution as the neuron. Each neuronal segment has a counterpart interstitial and a glial segment in which ionic release, diffusion and uptake, mediated by active mechanisms including Kir, a 3Na^+^/2K^+^ exchanger as well as Ca^2+^, Cl^−^ and Na^+^ pumps as well as passive diffusion, are calculated. Ion concentration changes are iteratively updated and integrated with the ionic driving forces^36^,^37^. The glial Kir in this model is based on the mathematical description^68^ where:


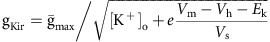


Here 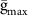 is the maximum conductance at a given [K^+^]_o_, *V*_m_ is the membrane potential, *V*_h_ shifts the opening midpoint potential −10 mV from *E*_K_ (–97 mV at [K^+^]_o_=3.5 mM) and *V*_s_ defines the steepness of the voltage dependence. In the mod file, *V*_s_ was set to 4 mV, representing six elementary charges moving across the entire electric field.

The model was mostly unchanged and run as published at ModelDB^36^, except that spatial segmentation was increased 10-fold to obtain 3-μm segments. To mimic different intercellular distances, we varied *α*, representing the fraction of extracellular volume divided by intracellular volume, between 0.005 and 1.0 (Supplementary Fig. 6c). For clarity, these values were expressed as intercellular distance (*δ*) between glia and the soma (with a diameter of 10 μm) and thus ranged between 12 and 2,000 nm. Final simulations were run with an interstitial volume fraction (*α*) of 0.045. To mimic glial Kir4.1 reduction, we adjusted Kir between 0.001 pS μm^−2^ (Ba^2+^ block) and 0.10 pS μm^−2^ (control density). Current injections were of short duration and ranged between 0 and 110 pA. The temperature in the simulations was nominally set to 33 °C and simulation time steps (d*t*) set to 25 μs.

### Statistics

Data are given as mean±s.e.m and the number of experiments and the corresponding statistical test is indicated. Data were statistically analysed with the built-in tools of Prism 6 (GraphPad Software Inc) and SPSS (v.23, IBM Corp, Armonk, NY, USA). When *n*⩾6, data were tested for normal distribution with a Shapiro–Wilk test and when positive, we applied a paired or unpaired *t*-test subsequently. Not normally distributed data or experiments with *n*<6 were tested with non-parametric Mann–Whitney test when data were unpaired or with Wilcoxon signed-rank test when experiments were paired. Comparisons between more than two groups were performed by a Kruskal–Wallis test or ANOVA where appropriate.

## Additional information

**How to cite this article:** Battefeld, A. *et al*. Myelinating satellite oligodendrocytes are integrated in a glial syncytium constraining neuronal high-frequency activity. *Nat. Commun.* 7:11298 doi: 10.1038/ncomms11298 (2016).

## Supplementary Material

Supplementary Information Supplementary Figures 1-7, Supplementary Tables 1-2 and Supplementary References.

Supplementary Movie 1 A 360° view of a confocal z-stack from a layer 5 neuron (red) and a s-OL (cyan) located at the soma base. Using contrast enhancement the processes of the s-OL were removed to show the close cell body apposition of the cells.

Supplementary Movie 2 A 360° view of the same cell pair as in Supplementary Movie 1 but with normal intensity settings. In this video the processes of the s-OL are visible around the neuron.

## References

[b1] Henneberger C., Papouin T., Oliet S. H. R. & Rusakov D. A. Long-term potentiation depends on release of D-serine from astrocytes. Nature 463, 232–236 (2010). 20075918 10.1038/nature08673 PMC2807667

[b2] Min R. & Nevian T. Astrocyte signaling controls spike timing-dependent depression at neocortical synapses. Nat. Neurosci. 15, 746–753 (2012). 22446881 10.1038/nn.3075

[b3] Chen J. . Heterosynaptic long-term depression mediated by ATP released from astrocytes. Glia 61, 178–191 (2013). 23044720 10.1002/glia.22425

[b4] Morquette P. . An astrocyte-dependent mechanism for neuronal rhythmogenesis. Nat. Neurosci. 18, 844–854 (2015). 25938883 10.1038/nn.4013

[b5] Tong X. . Astrocyte Kir4.1 ion channel deficits contribute to neuronal dysfunction in Huntington's disease model mice. Nat. Neurosci. 17, 694–703 (2014). 24686787 10.1038/nn.3691 PMC4064471

[b6] Wang F. . Astrocytes modulate neural network activity by Ca^2^^+^-dependent uptake of extracellular K+. Sci. Signal. 5, ra26 (2012). 22472648 10.1126/scisignal.2002334 PMC3515082

[b7] Vit J.-P., Ohara P. T., Bhargava A., Kelley K. & Jasmin L. Silencing the Kir4.1 potassium channel subunit in satellite glial cells of the rat trigeminal ganglion results in pain-like behavior in the absence of nerve injury. J. Neurosci. 28, 4161–4171 (2008). 18417695 10.1523/JNEUROSCI.5053-07.2008 PMC2533133

[b8] Baalman K. . Axon initial segment-associated microglia. J. Neurosci. 35, 2283–2292 (2015). 25653382 10.1523/JNEUROSCI.3751-14.2015 PMC4315845

[b9] Peters A., Palay S. L. & Webster H. D. The Fine Structure of the Nervous System: Neurons and their Supporting Cells 3, Oxford Univ. Press (1991).

[b10] Takasaki C. . Cytochemical and cytological properties of perineuronal oligodendrocytes in the mouse cortex. Eur. J. Neurosci. 32, 1326–1336 (2010). 20846325 10.1111/j.1460-9568.2010.07377.x

[b11] Kruger L. & Maxwell D. S. Electron microscopy of oligodendrocytes in normal rat cerebrum. Am. J. Anat. 118, 411–435 (1966). 5917194 10.1002/aja.1001180207

[b12] Ludwin S. K. The perineuronal satellite oligodendrocyte. a role in remyelination. Acta Neuropathol. 47, 49–53 (1979). 463504 10.1007/BF00698272

[b13] del Río Hortega P. Tercera aportación al conocimiento morfológico e interpretación funcional de la oligodendroglía *Mem. Real Soc. Esp. Hist. Nat*. **14**, 5–122 ((1928).

[b14] Szuchet S. . The genetic signature of perineuronal oligodendrocytes reveals their unique phenotype. Eur. J. Neurosci. 34, 1906–1922 (2011). 22132705 10.1111/j.1460-9568.2011.07922.x PMC4286392

[b15] Butt A. M. in Neuroglia eds Kettenmann H., Ransom B. R. 62–73Oxford Univ. Press (2013).

[b16] Nave K.-A. Myelination and support of axonal integrity by glia. Nature 468, 244–252 (2010). 21068833 10.1038/nature09614

[b17] Taniike M. . Perineuronal oligodendrocytes protect against neuronal apoptosis through the production of lipocalin-type prostaglandin D synthase in a genetic demyelinating model. J. Neurosci. 22, 4885–4896 (2002). 12077186 10.1523/JNEUROSCI.22-12-04885.2002 PMC6757748

[b18] Kole M. H. P. & Stuart G. J. Signal processing in the axon initial segment. Neuron 73, 235–247 (2012). 22284179 10.1016/j.neuron.2012.01.007

[b19] Kole M. H. P. First node of Ranvier facilitates high-frequency burst encoding. Neuron 71, 671–682 (2011). 21867883 10.1016/j.neuron.2011.06.024

[b20] Beltramo R. . Layer-specific excitatory circuits differentially control recurrent network dynamics in the neocortex. Nat. Neurosci. 16, 227–234 (2013). 23313909 10.1038/nn.3306

[b21] Bergles D. E., Roberts J. D., Somogyi P. & Jahr C. E. Glutamatergic synapses on oligodendrocyte precursor cells in the hippocampus. Nature 405, 187–191 (2000). 10821275 10.1038/35012083

[b22] Káradóttir R., Cavelier P., Bergersen L. H. & Attwell D. NMDA receptors are expressed in oligodendrocytes and activated in ischaemia. Nature 438, 1162–1166 (2005). 16372011 10.1038/nature04302 PMC1416283

[b23] Gipson K. & Bordey A. Analysis of the K+ current profile of mature rat oligodendrocytes in situ. J. Membr. Biol. 189, 201–212 (2002). 12395285 10.1007/s00232-002-1014-8

[b24] Hallermann S., de Kock C. P. J., Stuart G. J. & Kole M. H. P. State and location dependence of action potential metabolic cost in cortical pyramidal neurons. Nat. Neurosci. 15, 1007–1014 (2012). 22660478 10.1038/nn.3132

[b25] Maldonado P. P., Vélez-Fort M., Levavasseur F. & Angulo M. C. Oligodendrocyte precursor cells are accurate sensors of local K+ in mature gray matter. J. Neurosci. 33, 2432–2442 (2013). 23392672 10.1523/JNEUROSCI.1961-12.2013 PMC6619152

[b26] Djukic B., Casper K. B., Philpot B. D., Chin L.-S. & McCarthy K. D. Conditional knock-out of Kir4.1 leads to glial membrane depolarization, inhibition of potassium and glutamate uptake, and enhanced short-term synaptic potentiation. J. Neurosci. 27, 11354–11365 (2007). 17942730 10.1523/JNEUROSCI.0723-07.2007 PMC6673037

[b27] Kalsi A. S., Greenwood K., Wilkin G. & Butt A. M. Kir4.1 expression by astrocytes and oligodendrocytes in CNS white matter: a developmental study in the rat optic nerve. J. Anat. 204, 475–485 (2004). 15198689 10.1111/j.0021-8782.2004.00288.x PMC1571318

[b28] Schirmer L. . Differential loss of KIR4.1 immunoreactivity in multiple sclerosis lesions. Ann. Neurol. 75, 810–828 (2014). 24777949 10.1002/ana.24168

[b29] Hibino H. . Inwardly rectifying potassium channels: their structure, function, and physiological roles. Physiol. Rev. 90, 291–366 (2010). 20086079 10.1152/physrev.00021.2009

[b30] Tress O. . Panglial gap junctional communication is essential for maintenance of myelin in the CNS. J. Neurosci. 32, 7499–7518 (2012). 22649229 10.1523/JNEUROSCI.0392-12.2012 PMC6703577

[b31] Wasseff S. K. & Scherer S. S. Cx32 and Cx47 mediate oligodendrocyte:astrocyte and oligodendrocyte:oligodendrocyte gap junction coupling. Neurobiol. Dis. 42, 506–513 (2011). 21396451 10.1016/j.nbd.2011.03.003 PMC3773476

[b32] Maglione M. . Oligodendrocytes in mouse corpus callosum are coupled via gap junction channels formed by connexin47 and connexin32. Glia 58, 1104–1117 (2010). 20468052 10.1002/glia.20991

[b33] Ransom B. R. & Kettenmann H. Electrical coupling, without dye coupling, between mammalian astrocytes and oligodendrocytes in cell culture. Glia 3, 258–266 (1990). 2144505 10.1002/glia.440030405

[b34] Menichella D. M. . Genetic and physiological evidence that oligodendrocyte gap junctions contribute to spatial buffering of potassium released during neuronal activity. J. Neurosci. 26, 10984–10991 (2006). 17065440 10.1523/JNEUROSCI.0304-06.2006 PMC6674647

[b35] Kole M. H. P. & Stuart G. J. Is action potential threshold lowest in the axon? Nat. Neurosci. 11, 1253–1255 (2008). 18836442 10.1038/nn.2203

[b36] Somjen G. G., Kager H. & Wadman W. J. Computer simulations of neuron-glia interactions mediated by ion flux. J. Comput. Neurosci. 25, 349–365 (2008). 18297383 10.1007/s10827-008-0083-9

[b37] Kager H., Wadman W. J. & Somjen G. G. Seizure-like afterdischarges simulated in a model neuron. J. Comput. Neurosci. 22, 105–128 (2007). 17053996 10.1007/s10827-006-0001-y

[b38] Hattox A. M. & Nelson S. B. Layer V neurons in mouse cortex projecting to different targets have distinct physiological properties. J. Neurophysiol. 98, 3330–3340 (2007). 17898147 10.1152/jn.00397.2007

[b39] van Landeghem F. K. H., Weiss T. & von Deimling A. Expression of PACAP and glutamate transporter proteins in satellite oligodendrocytes of the human CNS. Regul. Pept. 142, 52–59 (2007). 17346813 10.1016/j.regpep.2007.01.008

[b40] Vostrikov V. M., Uranova N. A. & Orlovskaya D. D. Deficit of perineuronal oligodendrocytes in the prefrontal cortex in schizophrenia and mood disorders. Schizophr. Res. 94, 273–280 (2007). 17566708 10.1016/j.schres.2007.04.014

[b41] Wang F., Xu Q., Wang W., Takano T. & Nedergaard M. Bergmann glia modulate cerebellar Purkinje cell bistability via Ca2+-dependent K+ uptake. Proc. Natl Acad. Sci. USA 109, 7911–7916 (2012). 22547829 10.1073/pnas.1120380109 PMC3356677

[b42] Kofuji P. & Newman E. A. Potassium buffering in the central nervous system. Neuroscience 129, 1045–1056 (2004). 15561419 10.1016/j.neuroscience.2004.06.008 PMC2322935

[b43] Holthoff K. & Witte O. W. Directed spatial potassium redistribution in rat neocortex. Glia 29, 288–292 (2000). 10642755 10.1002/(sici)1098-1136(20000201)29:3<288::aid-glia10>3.0.co;2-8

[b44] Pannasch U., Derangeon M., Chever O. & Rouach N. Astroglial gap junctions shape neuronal network activity. Commun. Integr. Biol. 5, 248–254 (2012). 22896785 10.4161/cib.19410 PMC3419107

[b45] Syková E. & Nicholson C. Diffusion in brain extracellular space. Physiol. Rev. 88, 1277–1340 (2008). 18923183 10.1152/physrev.00027.2007 PMC2785730

[b46] Poopalasundaram S. . Glial heterogeneity in expression of the inwardly rectifying K(+) channel, Kir4.1, in adult rat CNS. Glia 30, 362–372 (2000). 10797616 10.1002/(sici)1098-1136(200006)30:4<362::aid-glia50>3.0.co;2-4

[b47] Bockenhauer D. . Epilepsy, ataxia, sensorineural deafness, tubulopathy, and KCNJ10 mutations. N. Engl. J. Med. 360, 1960–1970 (2009). 19420365 10.1056/NEJMoa0810276 PMC3398803

[b48] Neusch C., Rozengurt N., Jacobs R. E., Lester H. A. & Kofuji P. Kir4.1 potassium channel subunit is crucial for oligodendrocyte development and *in vivo* myelination. J. Neurosci. 21, 5429–5438 (2001). 11466414 10.1523/JNEUROSCI.21-15-05429.2001 PMC6762664

[b49] Haj-Yasein N. N. . Evidence that compromised K+ spatial buffering contributes to the epileptogenic effect of mutations in the human Kir4.1 gene (KCNJ10). Glia 59, 1635–1642 (2011). 21748805 10.1002/glia.21205

[b50] Griemsmann S. . Characterization of panglial gap junction networks in the thalamus, neocortex, and hippocampus reveals a unique population of glial cells. Cereb. Cortex 25, 3420–3433 (2014). 25037920 10.1093/cercor/bhu157 PMC4585496

[b51] Ma B. . Gap junction coupling confers isopotentiality on astrocyte syncytium. Glia 64, 214–226 (2016). 26435164 10.1002/glia.22924 PMC4595908

[b52] Kofuji P. . Kir potassium channel subunit expression in retinal glial cells: implications for spatial potassium buffering. Glia 39, 292–303 (2002). 12203395 10.1002/glia.10112

[b53] Pessia M., Tucker S. J., Lee K., Bond C. T. & Adelman J. P. Subunit positional effects revealed by novel heteromeric inwardly rectifying K+ channels. EMBO J. 15, 2980–2987 (1996). 8670799 PMC450239

[b54] Tanemoto M., Kittaka N., Inanobe A. & Kurachi Y. *In vivo* formation of a proton-sensitive K+ channel by heteromeric subunit assembly of Kir5.1 with Kir4.1. J. Physiol. 525, (Pt 3): 587–592 (2000). 10856114 10.1111/j.1469-7793.2000.00587.x PMC2269982

[b55] Zhang Y. . An RNA-sequencing transcriptome and splicing database of glia, neurons, and vascular cells of the cerebral cortex. J. Neurosci. 34, 11929–11947 (2014). 25186741 10.1523/JNEUROSCI.1860-14.2014 PMC4152602

[b56] Hamilton N. B., Kolodziejczyk K., Kougioumtzidou E. & Attwell D. Proton-gated Ca(2+)-permeable TRP channels damage myelin in conditions mimicking ischaemia. Nature 529, 523–527 (2016). 26760212 10.1038/nature16519 PMC4733665

[b57] Périer F., Radeke C. M. & Vandenberg C. A. Primary structure and characterization of a small-conductance inwardly rectifying potassium channel from human hippocampus. Proc. Natl Acad. Sci. USA 91, 6240–6244 (1994). 8016146 10.1073/pnas.91.13.6240 PMC44174

[b58] Xu N.-L. . Nonlinear dendritic integration of sensory and motor input during an active sensing task. Nature 492, 247–251 (2012). 23143335 10.1038/nature11601

[b59] Larkum M. A cellular mechanism for cortical associations: an organizing principle for the cerebral cortex. Trends Neurosci. 36, 141–151 (2013). 23273272 10.1016/j.tins.2012.11.006

[b60] Hirrlinger P. G. . Expression of reef coral fluorescent proteins in the central nervous system of transgenic mice. Mol. Cell. Neurosci. 30, 291–303 (2005). 16169246 10.1016/j.mcn.2005.08.011

[b61] Attwell D. & Iles J. F. Synaptic transmission: ion concentration changes in the synaptic cleft. Proc. Natl Acad. Sci. USA 206, 115–131 (1979). 10.1098/rspb.1979.0095 42066

[b62] Hardy R. J., Lazzarini R. A., Colman D. R. & Friedrich V. L. Cytoplasmic and nuclear localization of myelin basic proteins reveals heterogeneity among oligodendrocytes. J. Neurosci. Res. 46, 246–257 (1996). 8915902 10.1002/(SICI)1097-4547(19961015)46:2<246::AID-JNR13>3.0.CO;2-0

[b63] Bortone D. S., Olsen S. R. & Scanziani M. Translaminar inhibitory cells recruited by layer 6 corticothalamic neurons suppress visual cortex. Neuron 82, 474–485 (2014). 24656931 10.1016/j.neuron.2014.02.021 PMC4068343

[b64] Schindelin J. . Fiji: an open-source platform for biological-image analysis. Nat. Methods 9, 676–682 (2012). 22743772 10.1038/nmeth.2019 PMC3855844

[b65] van den Pol A. N. & Gorcs T. Synaptic relationships between neurons containing vasopressin, gastrin-releasing peptide, vasoactive intestinal polypeptide, and glutamate decarboxylase immunoreactivity in the suprachiasmatic nucleus: dual ultrastructural immunocytochemistry with gold-substituted silver peroxidase. J. Comp. Neurol. 252, 507–521 (1986). 2878014 10.1002/cne.902520407

[b66] Eldred W. D., Zucker C., Karten H. J. & Yazulla S. Comparison of fixation and penetration enhancement techniques for use in ultrastructural immunocytochemistry. J. Histochem. Cytochem. 31, 285–292 (1983). 6339606 10.1177/31.2.6339606

[b67] Korogod N., Petersen C. C. H. & Knott G. W. Ultrastructural analysis of adult mouse neocortex comparing aldehyde perfusion with cryo fixation. Elife 4, e05793 (2015). 10.7554/eLife.05793 PMC4530226 26259873

[b68] van Heukelom J. S. The role of the potassium inward rectifier in defining cell membrane potentials in low potassium media, analysed by computer simulation. Biophys. Chem. 50, 345–360 (1994).

