# Supplementary material for: Myelinating satellite oligodendrocytes are integrated in a glial syncytium constraining neuronal high-frequency activity
Source: Nat Commun. 2016 May 10;7:11298. doi: 10.1038/ncomms11298 (PMC4866043; doi:10.1038/ncomms11298)
Supplement: Supplementary Information — Supplementary Figures 1-7, Supplementary Tables 1-2 and Supplementary References. [file ncomms11298-s1.pdf]

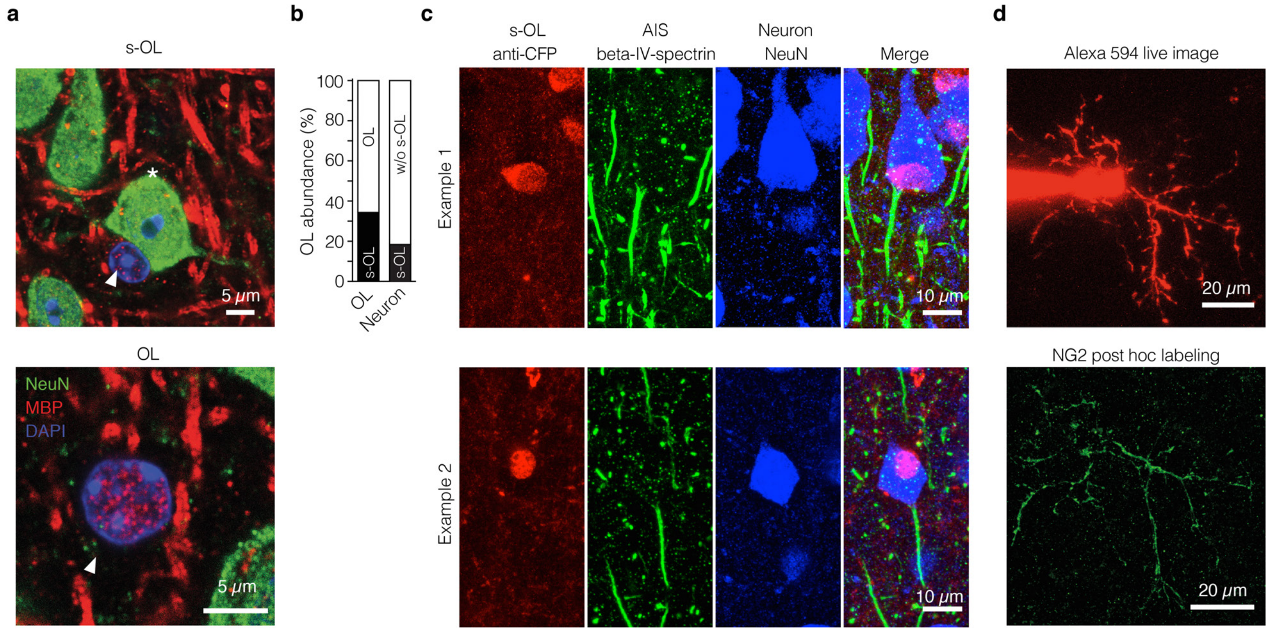

Supplementary Figure 1. Distribution analysis of s-OLs and experimental identification of OPCs.

(**a**) *Top*: Confocal image of a 40 μm slice that was immunohistochemically labeled with NeuN (green), MBP (red) and DAPI (blue). A s-OL (white arrowhead) can be seen at a NeuN positive soma (asterisk). *Bottom*: A non-satellite oligodendrocyte labeled with the same antibodies.

(**b**) Quantification of the s-OL distribution in layer 5 of the somatosensory cortex from images as shown in (a). NeuN positive neurons n = 2055; total oligodendrocytes n = 1103; neurons with s-OL n = 380; n = 3 animals, each 8 ROIs. Values are displayed as mean.

(**c**) Immunohistochemical co-labeling of CFP^+ oligodendrocytes, the AIS marker beta-IV-spectrin and the pan-neuron marker NeuN. The two representative examples of confocal z-projected images show the arrangement of s-OLs in relation to the AIS. Example 1 shows a typical s-OL that can be found closer to the AIS, but not on the AIS. In comparison in example 2 the s-OL is distal from the AIS. The edge of satellite OLs was on average 7.4 ± 1.4 μm away from the onset of the AIS as defined by the start of beta-IV-spectrin labeling (n = 26). When located at the neuron soma base the s-OLs were sometimes touching the AIS (6 out of 26), but oriented more to the somatic domain (see Supplementary Movie 1).

(d) *Top*: Satellite cell that was morphologically identified as OPC after filling with Alexa 594. *Bottom*: The cell shown in the live image was fixed with PFA after the recording and subsequently labeled for the OPC marker NG2 (polyclonal rabbit antibody, 1:250, AB5320, Millipore) that gave a positive signal.

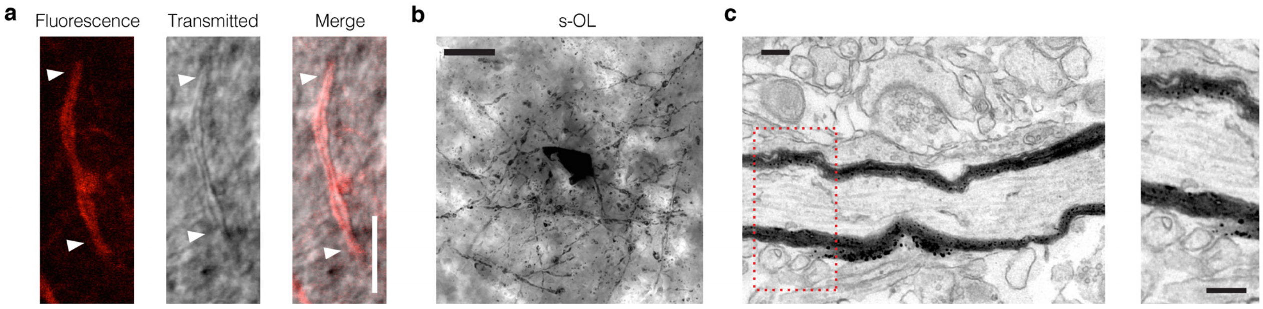

Supplementary Figure 2. Fluorescence and ultrastructure analysis of single identified s-OL internodes.

(**a**) A process of a single filled s-OL co-localizes with myelin structures that were identified by two dark bands in the transmitted light. Scale bar 10 μm.

(**b**) Maximum z-projected bright field image of a HRP filled s-OL after DAB reaction and embedding in epon. Several internodes and the cell body are visible as black staining. Scale bar 20 μm.

(**c**) *Left*: EM image of a longitudinal cut positive gold-labeled internode (black DAB product was substituted by colloidal gold particles). *Right*: Higher magnification image of the red box indicated in the left image. Scale bars 200 nm.

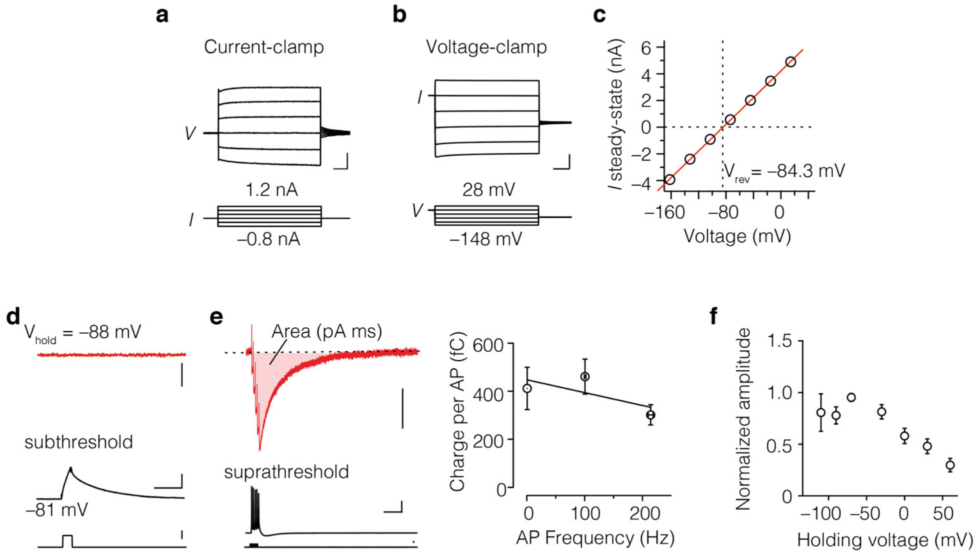

Supplementary Figure 3. Resting membrane characteristics of s-OLs and AP-evoked responses.

(**a**) Voltage responses of a s-OL to a family of hyperpolarizing and depolarizing current injections in current-clamp reveal an almost instantaneous and linear voltage response. Scale bars 5 mV and 0.1 s.

(**b**) Current responses elicited from 0 mV to voltage steps from –148 mV to 28 mV are large in amplitude and show a very rapid current onset. Scale bars 2 nA and 50 ms.

(**c**) The steady-state current was linear, reversed polarity at –84.3 mV and could be described by a linear fit (y = 0.04999*x + 4.21, red line). Plotted are mean ± SEM, n = 12 s-OLs, note that error bars are smaller than the symbols.

(**d**) Subthreshold current injection into a neuron did not induce an inward current in the s-OL (holding voltage of –88 mV). Traces are from the cell pair shown in Figure 3A and 3C. Scale bars 10 pA (top), 3 mV (middle) and 20 ms, 50 pA (bottom).

(**e**) *Left*: The charge per AP was determined as the total area of the s-OL inward current divided by the number of elicited APs. For 100 (n = 20) and 200 Hz (n = 5) a train of 5 APs was analyzed and for 1 Hz (n = 10) a single AP per s. The area (pA*ms) corresponds to the charge in fC. Scale bars 10 pA and 30 mV, 0.1 s. *Right*: Summary plot showing that the charge/AP is independent of AP frequency (Kruskal-Wallis test, P = 0.5). Data displayed as mean ± SEM.

(**f**) Inward current amplitudes of s-OLs in relation to different holding voltages normalized to the maximum recorded inward current (n = 9 cells). The inward current was strongly attenuated at more depolarized holding potentials, but was never completely abolished probably due to gap-junction coupling.

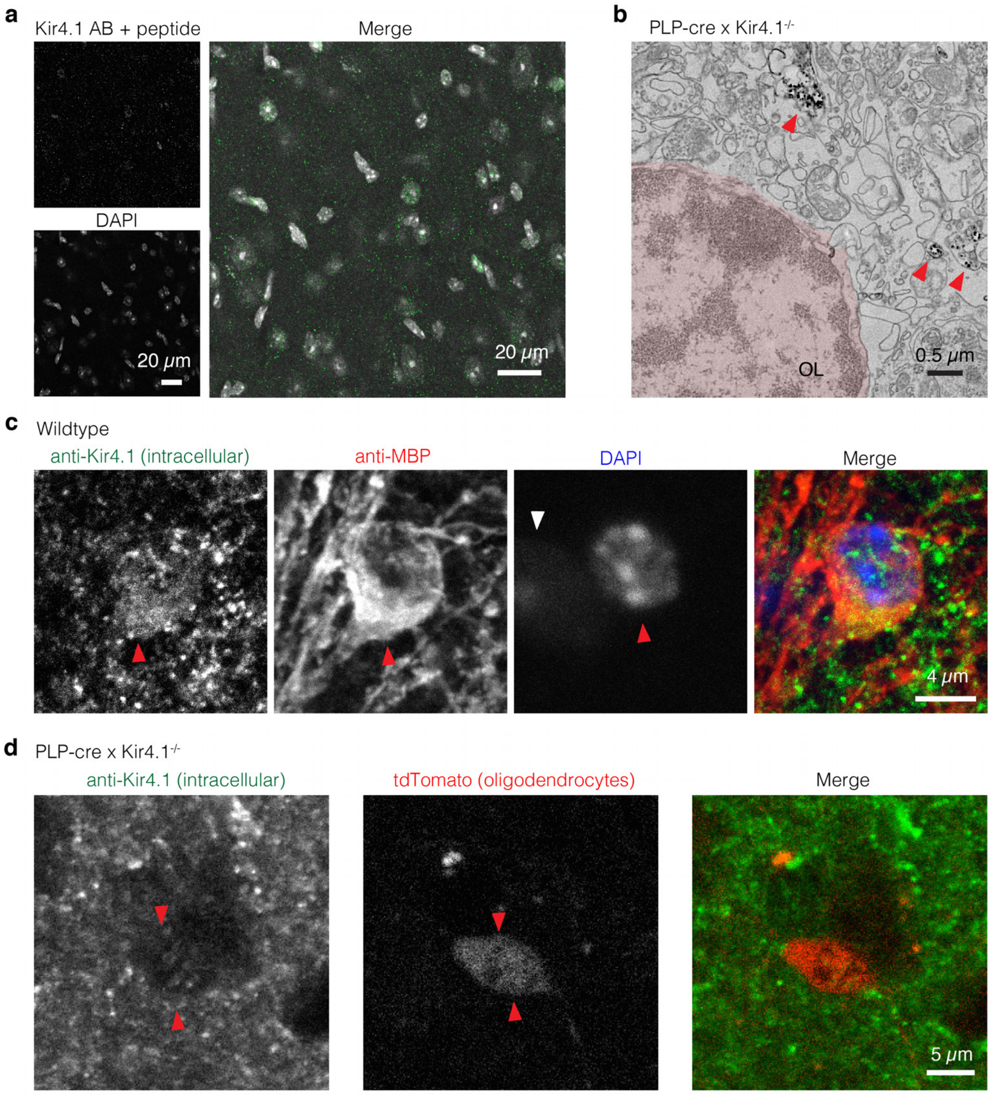

Supplementary Figure 4. Kir4.1 antibody labeling in neocortical tissue

(**a**) Specificity control for the Kir4.1 antibody (APC-035) after pre-incubation with the target peptide. We only observed a weak unspecific labeling that was not distinguishable from the background.

(**b**) EM image showing Kir4.1 immuno-gold particles (Kir4.1 antibody APC-035) confined to putative astrocytic processes but not in the OL (red) in the Kir4.1$^{-/-}$ mouse.

(**c**) Single plane confocal images of a combined immunolabeling of Kir4.1 (antibody APC-035), MBP and DAPI in a wildtype mouse. The oligodendrocyte (red arrow) exhibits a confined labeling at the cell body. Membrane areas that are not occupied by the nucleus show the strongest signal. Based on the DAPI labeling the OL was assumed to be a s-OL as its nucleus can be seen next to a larger cell nucleus, presumably from a neuron (white arrow).

(**d**) Single plane confocal images in the oligodendrocytic specific Kir4.1$^{-/-}$ mouse immunohistochemically labelled for Kir4.1 (Kir4.1 antibody APC-035) reveals no detectable signal of oligodendrocytes at their cell body.

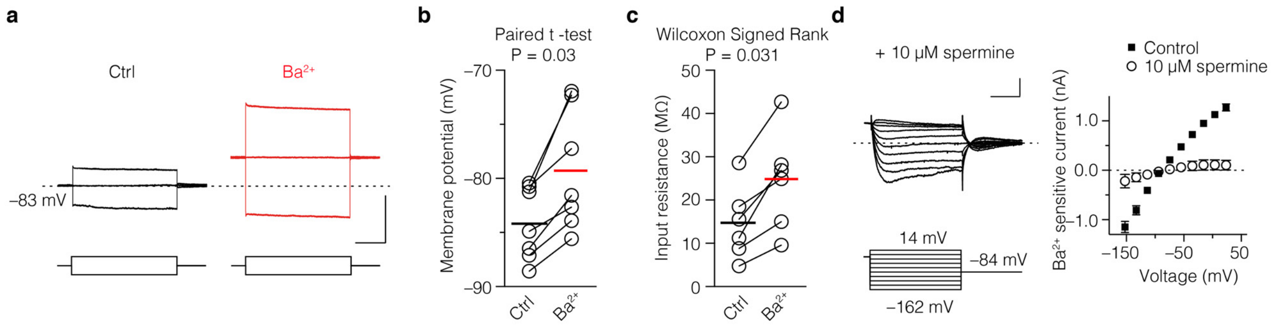

Supplementary Figure 5. Modulation of intrinsic s-OL membrane properties by Ba^{2+}

(**a**) Application of 100 μM Ba^{2+} depolarized the resting membrane potential and increased the input resistance. Voltage responses shown were evoked by 400 pA negative and positive current injections as shown below. Scale bars 20 mV and 0.2 s.

(**b**) Population data of the resting membrane potential before and after the application of Ba^{2+} reveals that all s-OLs depolarized in the presence of Ba^{2+} (n = 7). Horizontal lines (black – control; red – Ba^{2+}) indicate the mean of all paired experiments.

(**c**) Population data of the input resistance before and after Ba^{2+} application reveals a significant increase in Ba^{2+} (n = 6). Horizontal lines (black – control; red – Ba^{2+}) indicate the mean.

(**d**) *Left*: Ba^{2+} sensitive currents in the presence of 10 μM intracellular spermine were evoked with the voltage protocol shown below. Scale bars 0.1 nA and 100 ms. *Right*: Current-voltage relationship in the presence of spermine shows that outward rectification is strongly reduced, indicating a modulation of Kir channels by this polyamine. Rectifying ratio for s-OLs with spermine (+G/–G = 0.56 ± 0.18. n = 3) and for the control condition (+G/–G = 1.05 ± 0.21, n = 6, Fig. 5d). Data shown are mean ± SEM.

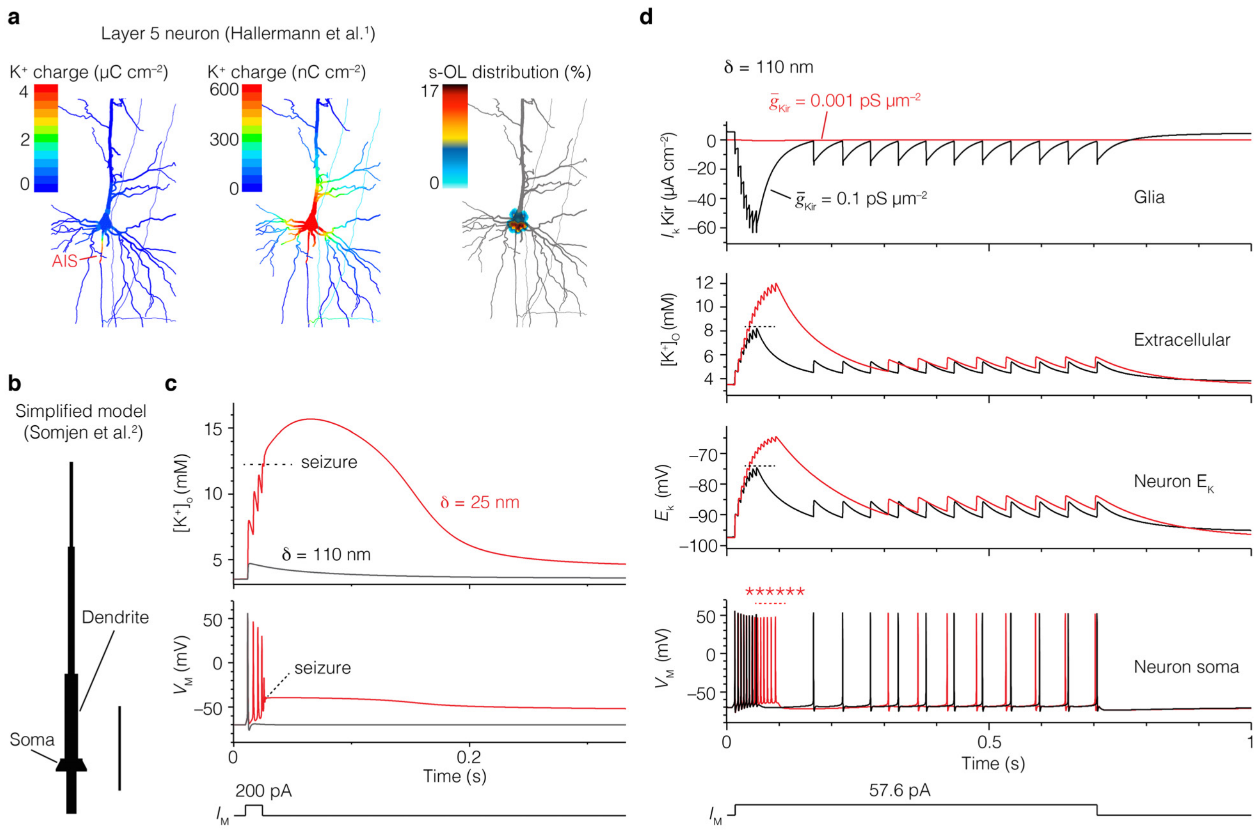

Supplementary Figure 6. Modeling $[K^+]_o$ and the role of glial Kir in modulation of neuronal excitability

(**a**) Space plot of the K^+ charge during the initiation and backpropagation of a single AP in a layer 5 pyramidal neuron model. For model details see reference[1]. *Left*: color range set to the maximum K^+ charge observed in the AIS, where high K^+ channel densities are expressed. *Middle*: color range set to a medium value to enable comparison of the somato-dendritic K^+ charge. *Right*: morphology of the same cell overlaid with the average distribution profile of s-OLs (as displayed in Figure 1). Note the large spatial overlap of neuronal K^+ efflux and s-OL location.

(**b**) Simplified morphology of the neuron used for neuron-glia simulations[2]. Scale bar 200 μm.

(**c**) With a 25 nm distance (or α = 0.01 uniform) the extracellular K^+ is poorly buffered causing seizure generation. Simulations were run with a Kir peak conductance density of 0.05 pS μm^{-2}.

(**d**) Simulations with a 110 nm intercellular distance (or α = 0.01 uniform) using a 700 ms current injection at 57.6 pA to compare model voltage responses. Reducing Kir conductance density from of 0.1 to 0.001 pS μm^{-2} primarily reduces the K^+ uptake during the high-frequency AP generation but has little to no impact on AP-evoked K^+ release during low-frequency steady firing (~10 Hz).

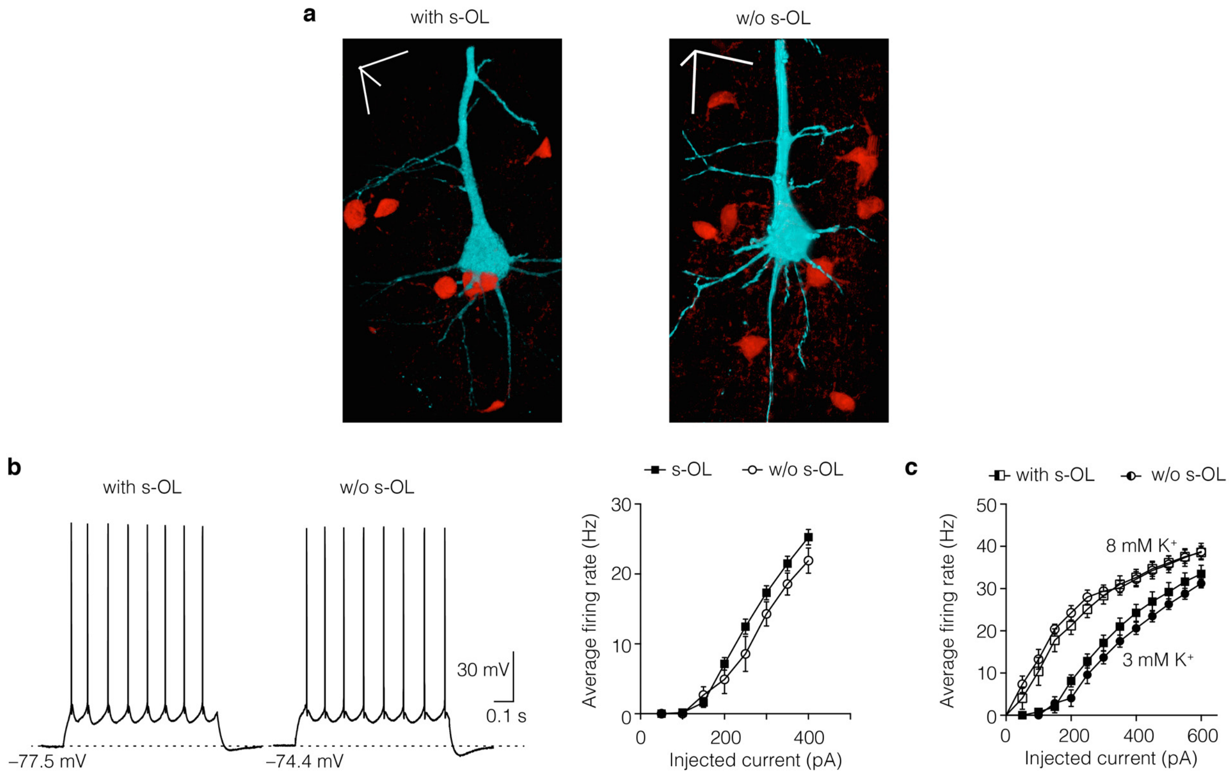

Supplementary Figure 7. Spatial arrangement of layer 5 neurons and oligodendrocytes and lack of impact on regular firing.

(**a**) 3D views from confocal z-stacks for the neurons displayed in Figure 7 illustrating the steric layout of neurons (cyan) and oligodendrocytes (red). Scale bars in all three dimensions 20 μm. The displayed images were generated with the 3D viewer plugin (FIJI).

(**b**) *Left*: Examples of regular AP firing patterns comparing two neurons with and without a satellite oligodendrocyte. *Right*: The *F-I* curve of the two populations shows neither difference in the rheobase (with s-OL 134.2 ± 9.0 pA, n = 25, vs. without s-OL 155.9 ± 24.5 pA, n = 9, unpaired t-test P = 0.31) nor the slope (with s-OL 0.1 ± 0.003 Hz pA^{-1}, n = 25, vs. without s-OL 0.098 ± 0.005 Hz pA^{-1}, n = 9, unpaired t-test P = 0.80). No difference of the firing rate was found between the two groups for the same current injection step (ANOVA post-hoc Bonferoni, P = 0.20).

(**c**) Summary plot of the average firing rate obtained from paired experiments in 3 mM K^+ and 8 mM K^+ for neurons with and without s-OL (n = 7 each group, see data in Figure 8). As expected by the application of high K^+ the *F-I* curve is shifted leftward.

Supplementary Table 1: Cell resting membrane properties

	V_m (mV)	R_N (MΩ)	G_m (nS)
L5 Neuron	–79.1 ± 0.1 (n = 32)	68.3 ± 5.0 (n = 50)	–
Grey matter s-OLs	–85.9 ± 0.6 (n = 101)	14.8 ± 1.9 (n = 63)	34.5 ± 2.1 (n = 39)
White matter OLs	–88.8 ± 0.9 (n = 4)	–	35.9 ± 3.9 (n = 4)
Astrocytes	–91.3 ± 0.9 (n = 18)	10.5 ± 2.4 (n = 15)	70 ± 20 (n = 4)
s-OLs Kir4.1 $^{-/-}$	–86.9 ± 0.9 (n = 27)	11.5 ± 3.4 (n = 11)	32.3 ± 4.0 (n = 20)
s-OL vs. Kir4.1 $^{-/-}$ *Mann Whitney P =*	0.92	0.23	0.31
s-OL vs. Astrocytes Mann Whitney P =	**0.0001**	0.178	0.075

Supplementary Table 2: AP properties of mouse layer 5 neurons

	Regular			IB		
	With s-OL (n = 34)	Without s-OL (n = 8)	*P*	With s-OL (n = 6)	Without s-OL (n = 8)	*P*
Amplitude (mV)	95.7 ± 0.9	97.2 ± 1.5	0.44	96.9 ± 2.4	95.7 ± 1.8	0.83
Half-width (*μ*s)	628 ± 16	644 ± 33	0.38	577 ± 28	623 ± 37	0.33
Afterdepolarization (mV)	–4.6 ± 0.5	–4.4 ± 1.1	0.90	–0.68 ± 0.82	–0.76 ± 2.09	0.63
dV/dt (V s^{-1})	577 ± 12	578 ± 24	0.99	592 ± 31	600 ± 25	0.81
V_m (mV)	–79.0 ± 0.5	–77.5 ± 0.6	0.20	–79.1 ± 1.0	–75.3 ± 0.5	**0.005**
R_N *(MΩ)*	73.34 ± 5.7	50.76 ± 2.5	**0.006**	49.09 ± 7.3	56.48 ± 5.6	0.94

Data are displayed as mean ± SEM.

Supplementary References

1. Hallermann, S., de Kock, C. P. J., Stuart, G. J. & Kole, M. H. P. State and location dependence of action potential metabolic cost in cortical pyramidal neurons. *Nat Neurosci* **15,** 1007–1014 (2012).
2. Somjen, G. G., Kager, H. & Wadman, W. J. Computer simulations of neuron-glia interactions mediated by ion flux. *J Comput Neurosci* **25,** 349–365 (2008).
